# Omega-3 fatty acids attenuate cardiovascular effects of short-term exposure to ambient air pollution

**DOI:** 10.1186/s12989-022-00451-4

**Published:** 2022-02-09

**Authors:** Hao Chen, Siqi Zhang, Wan Shen, Claudia Salazar, Alexandra Schneider, Lauren H. Wyatt, Ana G. Rappold, David Diaz-Sanchez, Robert B. Devlin, James M. Samet, Haiyan Tong

**Affiliations:** 1 Oak Ridge Institute for Science Education, Oak Ridge, TN USA; 2 Institute of Epidemiology, Helmholtz Zentrum München, Neuherberg, Germany; 3 Department of Public and Allied Health, Bowling Green State University, Bowling Green, OH USA; 4 Public Health and Integrated Toxicology Division, Center for Public Health and Environmental Assessment, Office of Research and Development, U.S. Environmental Protection Agency, 104 Mason Farm Rd, Chapel Hill, NC 27514 USA

**Keywords:** Omega-3 polyunsaturated fatty acids, Ambient air pollution, PM_2.5_, O_3_, Cardiovascular

## Abstract

**Background:**

Exposure to air pollution is associated with elevated cardiovascular risk. Evidence shows that omega-3 polyunsaturated fatty acids (omega-3 PUFA) may attenuate the adverse cardiovascular effects of exposure to fine particulate matter (PM_2.5_). However, it is unclear whether habitual dietary intake of omega-3 PUFA protects against the cardiovascular effects of short-term exposure to low-level ambient air pollution in healthy participants. In the present study, sixty-two adults with low or high dietary omega-3 PUFA intake were enrolled. Blood lipids, markers of vascular inflammation, coagulation and fibrinolysis, and heart rate variability (HRV) and repolarization were repeatedly assessed in 5 sessions separated by at least 7 days. This study was carried out in the Research Triangle area of North Carolina, USA between October 2016 and September 2019. Daily PM_2.5_ and maximum 8-h ozone (O_3_) concentrations were obtained from nearby air quality monitoring stations. Linear mixed-effects models were used to assess the associations between air pollutant concentrations and cardiovascular responses stratified by the omega-3 intake levels.

**Results:**

The average concentrations of ambient PM_2.5_ and O_3_ were well below the U.S. National Ambient Air Quality Standards during the study period. Significant associations between exposure to PM_2.5_ and changes in total cholesterol, von Willebrand factor (vWF), tissue plasminogen activator, D-dimer, and very-low frequency HRV were observed in the low omega-3 group, but not in the high group. Similarly, O_3_-associated adverse changes in cardiovascular biomarkers (total cholesterol, high-density lipoprotein, serum amyloid A, soluable intracellular adhesion molecule 1, and vWF) were mainly observed in the low omega-3 group. Lag-time-dependent biphasic changes were observed for some biomarkers.

**Conclusions:**

This study demonstrates associations between short-term exposure to PM_2.5_ and O_3_, at concentrations below regulatory standard, and subclinical cardiovascular responses, and that dietary omega-3 PUFA consumption may provide protection against such cardiovascular effects in healthy adults.

**Supplementary Information:**

The online version contains supplementary material available at 10.1186/s12989-022-00451-4.

## Background

Ambient air pollution is a major global environmental health problem. Exposure to ambient air pollution [particulate matter (PM) and ozone (O_3_)] was estimated to be responsible for 4.51 million deaths worldwide in 2019 [1]. Among the health impacts of air pollution, cardiovascular diseases (CVD) garner great concern as ischemic heart disease and stroke are the top two leading causes of death worldwide [2]. Short-term and long-term exposure to fine PM (PM_2.5_) is linked to an elevated risk for myocardial infarction, stroke, heart failure, and arrhythmia, and potentiates development of chronic cardiometabolic conditions such as diabetes [3].

As oxidative stress and inflammation are important mechanistic pathways mediating air pollution-associated cardiovascular effects, research has been undertaken to investigate potential interventional strategies at an individual level, focusing on these pathways to confer cardiovascular protection. Some omega-3 polyunsaturated fatty acids (PUFA) are dietary fats from marine sources [4]. Mechanistically, marine omega-3 PUFA, eicosapentaenoic acid (EPA, 20:5) and docosahexaenoic acid (DHA, 22:6), can serve as antioxidants and are also substrates for the synthesis of specialized pro-resolving mediators (SPMs) that orchestrate key signaling processes in mediating the resolution of inflammation and a return to homeostasis [5, 6]. Evidence shows that dietary omega-3 PUFA intake in the dose range of 2–4 g/day as fish or fish-oil products is associated with 25–40% lower blood triglyceride and possibly reduced cardiovascular risk among CVD patients [7].

A few studies have reported health benefits of omega-3 PUFA against air pollution exposure. We previously showed that fish oil supplementation (3 g/day for 4 weeks) attenuated adverse cardiac and lipid effects associated with a 2-h exposure to concentrated ambient particulate matter (avg. 278 µg/m^3^) in healthy middle-aged participants in a controlled exposure study setting [8]. Lin and colleagues reported that fish oil supplementation (2.5 g/day for 2 months) blunted ambient PM_2.5_ (avg. 38 µg/m^3^)—induced changes in biomarkers of inflammation, coagulation, and endothelial function among young adults in China [9]. Fish oil supplementation also alleviated systemic oxidative stress caused by ambient O_3_ and nitrogen dioxide in the same participants [10]. It should be noted that the air pollutant levels in these studies were higher than the U.S. National Ambient Air Quality Standards (NAAQS). However, evidence has shown increased cardiovascular risk in populations exposed to air pollution at concentrations below the established air quality standards, especially in susceptible groups [11, 12]. Thus, knowledge gaps remain on whether increased dietary omega-3 PUFA consumption can attenuate cardiovascular effects caused by ambient air pollution at low levels.

In this panel study, participants were enrolled based on their habitual omega-3 PUFA dietary intake and stratified according to their erythrocyte omega-3 index. We focused on subclinical endpoints of blood lipids, vascular inflammation, coagulation and fibrinolysis, and heart rate variability (HRV) and repolarization. We hypothesized that habitual omega-3 PUFA consumption can alleviate adverse cardiovascular effects induced by short-term exposure to low levels of ambient PM_2.5_ and O_3_.

## Results

### Descriptive statistics

Of the 62 enrolled participants, the majority (56) completed 5 sessions, while 3 completed 4 sessions and another 3 completed 3 sessions. As shown in Table 1, 28 participants were in the low omega-3 group and 34 in the high group. No statistical differences were observed in age, sex, race, smoking history, BMI, or systolic and diastolic blood pressure between the two groups. As expected, the difference in omega-3 index between the low and high groups was statistically significant (4.0% vs. 6.8%, *p* < 0.001). The description of all cardiovascular biomarkers across all sessions in both low and high omega-3 groups are presented in Additional file 1: Table S1.

**Table 1:** Participant characteristics

	Low omega-3 (*n* = 28)	High omega-3 (*n* = 34)	All (*n* = 62)
Age (years), mean (SD)	37 (8)	40 (9)	38 (9)
Sex, n (%)			
Male	10 (35.7)	13 (38.2)	23 (37.1)
Female	18 (64.3)	21 (61.8)	39 (62.9)
Race, n (%)			
African American	9 (32.1)	5 (14.7)	14 (22.6)
Asian	0 (0)	3 (8.8)	3 (4.8)
Caucasian	19 (67.9)	26 (76.5)	45 (72.6)
Smoking history, n (%)			
Never-smoker	22 (78.6)	32 (94.1)	54 (87.1)
Ex-smoker	6 (21.4)	2 (5.9)	8 (12.9)
BMI (kg/m^2^), mean (SD)	24.9 (3.3)	24.4 (3.1)	24.6 (3.2)
Omega-3 index (%), mean (SD)	4.0 (0.8)	6.8 (1.2)*	5.5 (1.7)
SBP (mmHg), mean (SD)	113.0 (8.8)	109.9 (9.9)	111.3 (9.5)
DBP (mmHg), mean (SD)	71.5 (6.7)	69.5 (7.3)	70.4 (7.1)

Statistical difference between low and high omega-3 groups was derived using Kruskal–Wallis rank sum tests for continuous variables and Fisher’s exact tests for categorical variables, *p < 0.05 for the difference between groups. BMI, body mass index; DBP, diastolic blood pressure; SBP, systolic blood pressure; SD, standard deviation

During the study period, daily PM_2.5_ concentrations ranged from 1.8 to 68.0 µg/m^3^, with a mean of 10.2 µg/m^3^ and an IQR of 4.7 µg/m^3^. Average maximum 8-h O_3_ concentration was 40.8 ppb (range 10–71 ppb, IQR: 17 ppb). Temperature and relative humidity ranged from -8.6 to 31.1 °C and 30 to 100%, respectively. We observed weak or moderate correlations between air pollutants and meteorological measurements (Table 2).

**Table 2:** Distribution and correlation of air pollution concentrations and meteorological measurements during the study period (Oct. 6, 2016–Sep. 5, 2019)

	Mean (SD)	Range	IQR	Spearman correlation coefficient			
				PM_2.5_	O_3_	NO_2_	Temperature
PM_2.5_ (µg/m^3^)	10.2 (4.1)	1.8–68.0	4.7				
O_3__8h (ppb)	40.8 (11.1)	10–71	17	0.16			
NO_2_ (ppb)	5.3 (3.8)	0.8–24.2	3.8	0.45	− 0.13		
Temperature (°C)	16.5 (8.9)	− 8.6 to 31.1	15.2	− 0.10	0.47	− 0.42	
Relative humidity (%)	70.2 (15.6)	30–100	22.2	− 0.19	− 0.46	− 0.21	0.17

IQR, interquartile range; NO_2_, nitrogen dioxide; O_3_, ozone; PM, particulate matter; SD, standard deviation

### Overview of the regression results

As summarized in Table 3, differential effects of ambient air pollution on cardiovascular biomarkers were observed in the low and high omega-3 groups. Specifically, in the low omega-3 group, significant associations were observed between increased air pollutant concentrations and changes in cardiovascular biomarkers. However, in the high omega-3 group, the associations were either null or in a direction of mitigation of the adverse effects. The detailed results are described below. We only report effect estimates [95% confidence interval (CI)] for markers significantly associated with either PM_2.5_ or O_3_ and *p*_interaction_ if between-group difference was significant (*p*_interaction_ < 0.1).

**Table 3:** Summary of associations between air pollutant and cardiovascular biomarkers in low and high omega-3 groups

Outcome	PM_2.5_		O_3_	
	Low omega-3	High omega-3	Low omega-3	High omega-3
Blood lipids				
Total cholesterol	↓_*L*1−2 *and* 5*dMA*_	→	↑_L0_, ↓_L2-4_	↓_*L*1_
HDL	→	→	↓_*L*2−4 *and* 5*dMA*_	→
LDL	→	→	→	↓_*L*1_
Vascular inflammation				
SAA	→	→	↑_*L*1 *and* 5*dMA*_	→
sICAM-1	→	→	↑_*L*0_	↓_*L*4_
sVCAM-1	→	→	→	→
Coagulation/fibrinolysis factors				
tPA	↑_*L*3_	→	→	→
vWF	↑_*L*0_, ↓_*L*3−4_	→	↑_*L*0_	→
D-dimer	↑_*L*1_, ↓_*L*4_	→	→	→
HRV and repolarization				
VLF	↓_*L*0_	→	→	→
P complexity	→	↑_*L*4 *and* 5*dMA*_	→	→

Arrows “↓, ↑, and →” indicate negative, positive and null associations between air pollutant and cardiovascular biomarker, respectively. 5dMA, 5-day moving average; HDL, high density lipoprotein; HRV, heart rate variability; L0, lag0; L1, lag1; L2, lag2; L3, lag3; L4, lag4; LDL, low density lipoprotein; O_3_, ozone; PM, particulate matter; SAA, serum amyloid A; sICAM-1, soluble intercellular adhesion molecule 1; tPA, tissue plasminogen activator; VLF, very-low frequency; vWF, von Willebrand factor

### Effects of blood omega-3 PUFA on the association between PM_2.5_ exposure and cardiovascular biomarkers

**Fig. 1:**
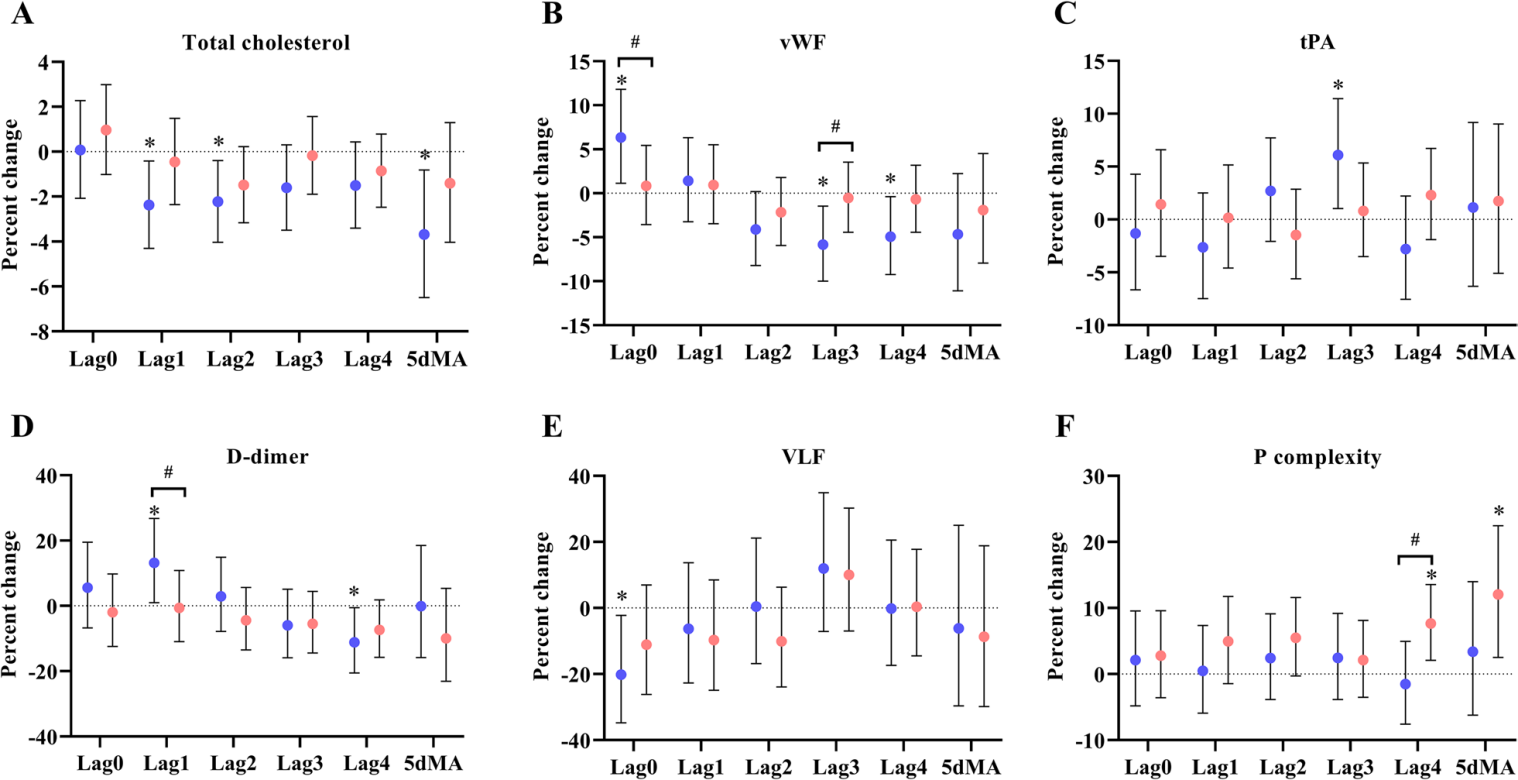
Influence of omega-3 PUFA on PM_2.5_ affected cardiovascular biomarkers. Effect estimates (95% CI) in total cholesterol (**A**), vWF (**B**), tPA (**C**), D-dimer (**D**), VLF (**E**), and P complexity (**F**) were corresponded to an IQR increase in PM_2.5_ concentrations in the low (blue) and high (red) omega-3 groups. * *p* < 0.05 for significant association within a group. ^#^ *p*_interaction_ < 0.1 and ^##^ *p*_interaction_ < 0.05 for significant differences in the effect estimates between groups

The significant effects of PM_2.5_ on total cholesterol, vWF, tPA, D-dimer, and VLF were observed in the low omega-3 group, but not in the high group (Table 3, Fig. 1). Specifically, in the low omega-3 group: an IQR increase in the concentration of PM_2.5_ was associated with decreased total cholesterol at lag1 [-2.4% (-4.3%, -0.4%)], lag2 [-2.2% (-4.0%, -0.4%)], and 5dMA [-3.7% (-6.5%, -0.8%)] (Fig. 1A); PM_2.5_ was positively associated with vWF at lag0 [6.3% (1.1%, 11.8%), *p*_*interaction*_ = 0.099] while the association shifted to negative at lag3 [-5.8% (-10.0%, -1.5%), *p*_*interaction*_ = 0.07] and lag4 [-4.9% (-9.2%, -0.4%)] (Fig. 1B); PM_2.5_ was associated with increased tPA at lag3 [6.1% (1.0%, 11.4%)] (Fig. 1C); PM_2.5_ was associated with increased D-dimer at lag1 [13.1%, (1.0%, 26.8%), *p*_*interaction*_ = 0.09] but the association was negative at lag4 [-11.1% (-20.6%, -0.6%)] (Fig. 1D); PM_2.5_ was also associated with decreased VLF at lag0 [-20.2% (-34.8%, -2.2%)] (Fig. 1E). These associations were not observed in the high omega-3 group. In the high omega-3 group, positive associations between PM_2.5_ and P complexity were observed at lag4 [7.7% (2.1%, 13.6%), *p*_*interaction*_ = 0.03] and 5dMA [12.0% (2.5%, 22.5%)] (Fig. 1F). No significant effects of PM_2.5_ on other biomarkers were observed in either group (Additional file 1: Tables S2-S6).

### Effects of blood omega-3 PUFA on the association between O_3_ exposure and cardiovascular biomarkers

**Fig. 2:**
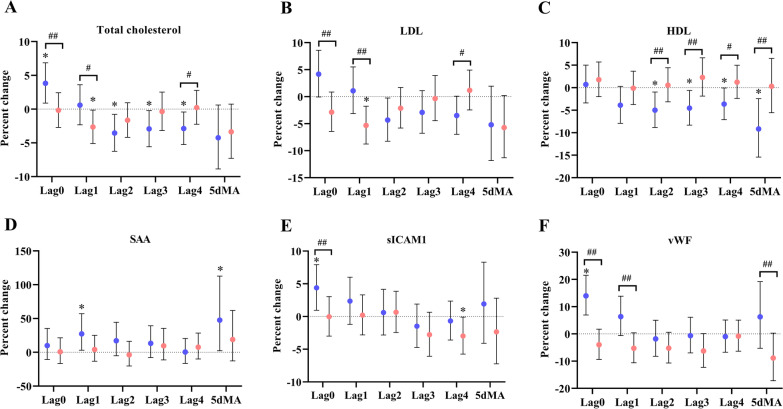
Influence of omega-3 PUFA on O_3_ affected cardiovascular biomarkers. Effect estimates (95% CI) in total cholesterol (**A**), LDL (**B**), HDL (**C**), SAA (**D**), sICAM-1 (**E**), and vWF (**F**) were corresponded to an IQR increase in O_3_ concentrations in the low (blue) and high (red) omega-3 groups. *p < 0.05 for significant association within a group. ^#^*p*_interaction_ < 0.1 and ^##^*p*_interaction_ < 0.05 for significant differences in the effect estimates between groups

Similar to the findings with PM_2.5_ exposure, significant associations between O_3_ exposure and the biomarkers were observed in the low omega-3 group with only a few found in the high group (Table 3, Fig. 2). In the low omega-3 group: an IQR increase in the concentration of O_3_ was significantly associated with increases in total cholesterol at lag0 [3.8% (0.9%, 6.9%), *p*_*interaction*_ = 0.02] but the association was negative at lag2 [-3.5% (-6.2%, -0.8%)], lag3 [-2.9% (-5.6%, -0.2%)], and lag4 [-2.9% (-5.2%, -0.4%)] (Fig. 2A); O_3_ exposure was significantly associated with decreases in HDL at lag2 [-5.0% (-8.8%, -1.0%), *p*_*interaction*_ = 0.03], lag3 [-4.5% (-8.3%, -0.6%), *p*_*interaction*_ = 0.01], lag4 [-3.6% (-7.1%, -0.1%), *p*_*interaction*_ = 0.05], and 5dMA [-9.2% (-15.4%, -2.4%), *p*_*interaction*_ = 0.01] (Fig. 2C); O_3_ exposure was significantly associated with increases in SAA at lag1 [27.2% (3.1%, 57.0%)] and 5dMA [47.5% (2.3%, 112.7%)] (Fig. 2D); O_3_ exposure was also associated with increases in sICAM-1 [4.4% (1.0%, 7.9%), *p*_*interaction*_ = 0.03] and vWF [14.0% (6.9%, 21.5%), *p*_*interaction*_ < 0.01)] at lag0 (Figs. 2E and 2F). In the high omega-3 group, a negative association was observed between O_3_ and total cholesterol [-2.7% (-5.1%, 0.1%), *p*_*interaction*_ = 0.07] and between O_3_ and LDL [-5.3% (-8.8%, -1.8%), *p*_*interaction*_ = 0.01] at lag1 (Figs. 2A and 2B). We did not observe significant associations between O_3_ exposure and other cardiovascular biomarkers in either group (Additional file 1: Table S2-S6).

**Fig. 3:**
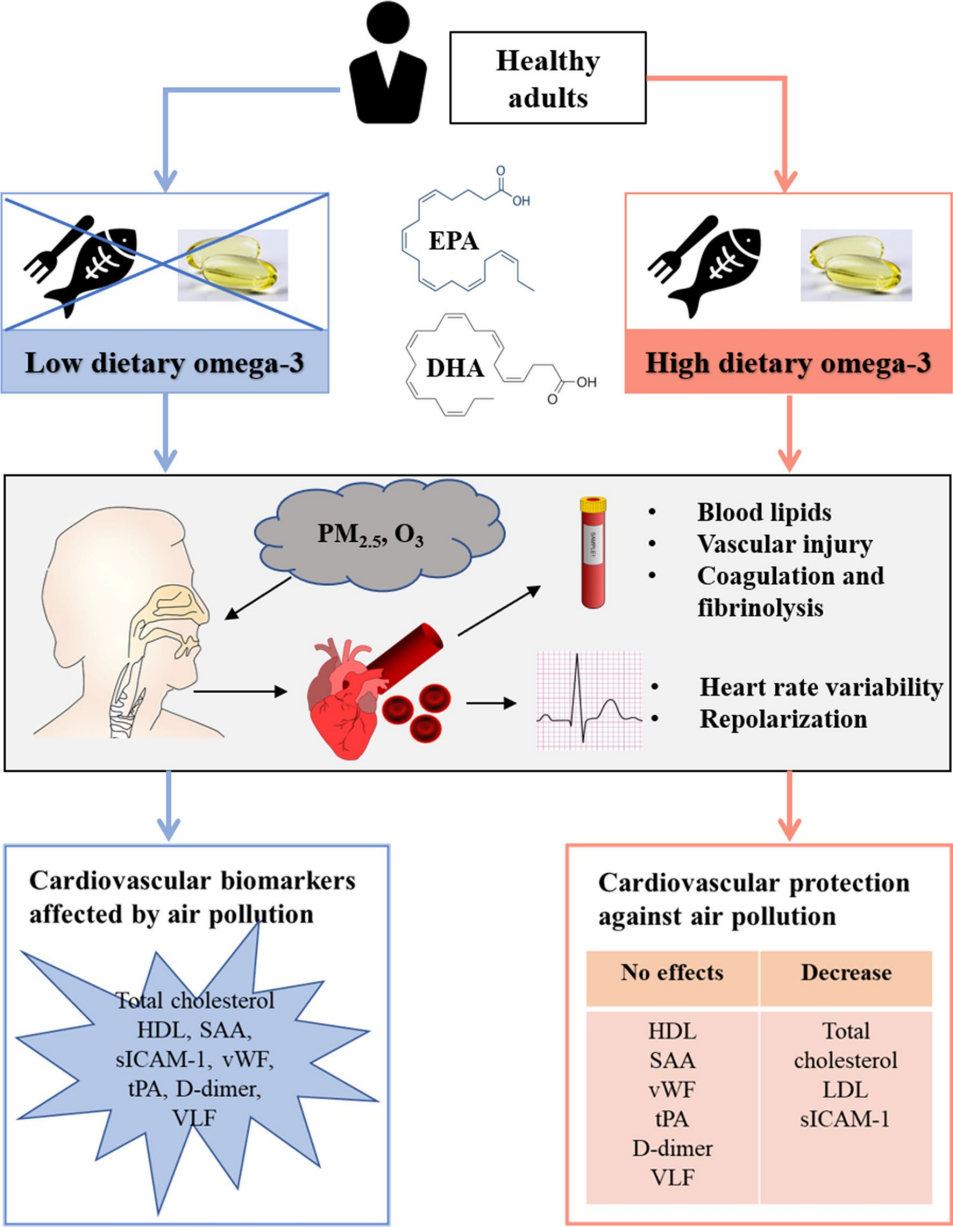
Schematic showing cardiovascular benefits of dietary omega-3 PUFA against short-term exposure to ambient air pollution. Healthy participants were enrolled in the low and high omega-3 groups based on their dietary omega-3 PUFA intake. Associations between exposure to ambient PM_2.5_ and O_3_ and cardiovascular biomarkers in blood and heart rate variability were assessed. Differential impacts of dietary omega-3 PUFA were observed on the cardiovascular biomarkers in response to short-term exposure to low-level ambient air pollution. DHA, docosahexaenoic acid; EPA, eicosapentaenoic acid; HDL, high-density lipoprotein; LDL, low-density lipoprotein; PM_2.5_, fine particulate matter; SAA, serum amyloid A; sICAM-1, soluble intercellular adhesion molecule 1; tPA, tissue plasminogen activator; VLF, very-low frequency; vWF, von Willebrand factor

All effect estimates remained stable in two pollutant models (Additional file 1: Table S7–S8) and after excluding outliers (Additional file 1: Table S9), indicating that the air pollutants act independently and that the effect estimates are not likely to be driven by outcome outliers.

## Discussion

In the present study, we investigated the potential cardioprotective effects of dietary omega-3 PUFA consumption against short-term exposure to low levels of ambient air pollution in healthy adults. As summarized in Fig. 3, we examined a range of cardiovascular biomarkers in response to exposure to ambient PM_2.5_ and O_3_, and report protective effects of higher omega-3 PUFA levels in mitigating changes in blood lipids, vascular inflammation, coagulation and fibrinolysis, and HRV.

Although the average concentrations of ambient air pollution during this study period were well below the U.S. NAAQS (24-h PM_2.5_: 35 μg/m^3^; 8-h O_3_: 70 ppb), significant changes in cardiovascular biomarkers were observed in association with these short-term exposures. Similarly, several studies have reported short-term exposure to ambient PM_2.5_ below the NAAQS levels was associated with cardiovascular effects in susceptible individuals in the U.S. [13–15]. In addition, some studies showed that long-term exposure to PM_2.5_, PM_10_, or NO_2_ at concentrations below established air quality standards was significantly associated with increased cardiovascular and respiratory risk among susceptible populations [11, 12]. These findings highlight the need for research on the low-level air pollution-induced health effects and potential interventions that may be effective against such low-level exposures.

Previous efforts have been made to identify interventional approaches to reduce the adverse health effects of air pollution [16]. Mechanistically, air pollution induced-cardiovascular effects are partly driven by oxidative stress and inflammation, suggesting an approach using dietary supplementation with antioxidant and anti-inflammatory agents [17]. Marine omega-3 PUFA are rich in oxidizable carbon–carbon double bonds and are substrates for the synthesis of SPMs. These features of omega-3 PUFA confer antioxidant and anti-inflammatory properties and therefore may blunt the pathophysiology of arteriosclerosis and acute coronary syndrome [4]. A meta-analysis of 13 clinical trials reported a consensus that omega-3 PUFA supplementation lowers the risk for myocardial infarction, coronary heart disease and CVD [18]. Two controlled randomized trials have demonstrated that dietary fish oil supplementation provided cardioprotective effects against high levels of PM_2.5_ exposure [8, 9]. Our results are in line with these studies in that we show omega-3 PUFA can mitigate adverse cardiovascular effects caused by exposure to air pollution.

Elevated blood lipids, especially triglycerides, total cholesterol and LDL are risks factors for CVD, while increased HDL is considered protective [19]. In the present study, decreased total cholesterol levels were observed in association with PM_2.5_ in the low omega-3 group. This result is in line with another study conducted in North Carolina showing that short-term exposure to ambient PM_2.5_ was associated with decreased blood lipid levels in diabetic patients [13]. However, it should be noted that both LDL and HDL, two important lipoproteins for cardiovascular risks, were not affected by PM_2.5_ regardless of omega-3 PUFA levels. Our previous controlled exposure study also reported that fish oil supplementation did not change the blood HDL and LDL associated with PM_2.5_ exposure [8], suggesting that dietary supplementary omega-3 PUFA did not significantly alter these lipoproteins. Triglyceride levels in the high omega-3 group showed a decreasing trend (Additional file 1: Table S1) that was not seen in the low omega-3 group, consistent with the use of fish oil supplementation for the treatment of hypertriglyceridemia [20].

Coagulation and fibrinolysis are two highly regulated and inter-related processes in response to tissue injury, ensuring balanced homeostasis of thrombus formation and degradation. vWF plays a major role in blood coagulation by binding to factor VIII and promoting platelet adhesion to injured vasculature [21]. tPA is a serine protease that converts plasminogen to plasmin for fibrin degradation, while D-dimer is a product of the fibrin degradation process [21, 22]. In the present study, elevated vWF was associated with immediate PM_2.5_ exposure (lag0), but the association shifted to null or negative at delayed lag days in the low omega-3 group, suggesting an acute PM_2.5_ impact on the increased coagulation activity. Consistently, a meta-analysis showed that a positive association between short-term PM_2.5_ exposure and vWF was only observed within 3 days of the exposure [23]. An association between PM_2.5_ exposure and elevated tPA or D-dimer in the low omega-3 group suggests an active fibrinolytic process in response to PM_2.5_ exposure. Interestingly, none of the significant findings mentioned above were found in the high omega-3 group, suggesting participants with high blood omega-3 PUFA levels were less susceptible to PM_2.5_ induced coagulation and fibrinolytic activities. Lin and colleagues also reported amelioration of plasma vWF and plasminogen by fish oil supplements in response to PM_2.5_ exposure in young subjects [9].

HRV is an index of autonomic nervous system control on the heart. A meta-analysis of 29 epidemiological studies showing that exposure to PM_2.5_ could alter HRV, including LF and HF [24]. Changes in VLF power, strongly correlated with normal sinus beats, have been shown to be associated with the risk for arrhythmic death [25]. In this study, a negative association between PM_2.5_ and VLF was observed in the low omega-3 group at lag0, implying possible effects on normal sinus beats associated with acute PM_2.5_ exposure. PM_2.5_-induced increases in P-wave complexity were observed at lag4 and 5dMA only in the high omega-3 group. As increased P-wave indices are associated with abnormal atrial conduction [26], our results suggest that omega-3 PUFA may affect atrial conduction with short-term exposure to PM_2.5_, although this finding warrants further investigation. Most of these changes were prominent in the low but not in the high omega-3 group, suggesting that omega-3 PUFA may modulate PM_2.5_-induced cardiac changes. However, caution is advised when interpreting the HRV results given that these healthy participants were exposed to low levels of ambient air pollution and the transient HRV changes may not be captured by a relatively short period of monitoring in this study.

Similarly, most adverse associations between O_3_ and cardiovascular biomarkers were in the low omega-3 group while the associations were either null or protective in the high omega-3 group. Specifically, most significant associations between blood lipids or vascular inflammation biomarkers and O_3_ exposure were observed in the low omega-3 group. The significant changes in total cholesterol and HDL in the low omega-3 group and LDL in the high omega-3 group, suggesting that dietary omega-3 PUFA may maintain a blood lipid profile in favor of reduced cardiac risk in response to O_3_ exposure. As O_3_ is considered a strong oxidant that can promote blood lipid oxidation [27], the quenching or antioxidant properties of omega-3 PUFA may mitigate such effects.

SAA is a biomarker of acute inflammation and tissue injury while sICAM-1 and sVCAM-1 participate in leukocyte adhesion to the endothelium and play an important role in all stages of atherosclerosis [28]. Increased SAA, sICAM-1 and sVCAM-1 levels in association with O_3_ were found in the low omega-3 group, but not in the high group, indicating the protection of omega-3 PUFA against O_3_-induced vascular inflammation. Data on the modifying effects of omega-3 PUFA on ozone-induced health effects are sparse. A study showed amelioration of fish oil on systemic oxidative stress induced by ambient O_3_ and NO_2_ exposure in human subjects [10]. An animal study also found vasoprotective effects and alleviation of cardiac dysfunction of fish oil supplementation against O_3_ exposure (800 ppb) in rats [29, 30].

It is noteworthy that we observed a lag-time-dependent biphasic change in several biomarkers in the low omega-3 group. Specifically, the positive associations between PM_2.5_ or O_3_ and total cholesterol, LDL, sICAM-1, vWF, and D-dimer were mainly observed at lag0 or lag1, but the associations trended null and negative at lag2-4. These observations indicate that the air pollutant-induced adverse effects are acute and normally reversible, which are consistent with current literature [31, 32]. The null and negative associations at delayed lag days suggest that the low-level of air pollution in our study did not have an extended adverse impact on the assessed biomarkers. This could be partly explained by the active defensive responses to limit and resolve the adverse effects in the subclinical biomarkers caused by low-level of air pollution. Omega-3 PUFA is an antioxidant that can readily react with oxidant air pollutants potentially mitigating their interaction with tissue targets. On the other hand, prolonged exposure to air pollution may generate reactive oxidized lipid products that are biologically active. We recently reported that a high omega-3 index protected lung function decrements associated with ozone exposure in the immediate term but potentiated the effect on lagging days [33]. Nevertheless, these changes were mainly observed in the low omega-3 group, implying that an increased susceptibility to adverse impacts of air pollution are in participants who are deficient in omega-3 PUFA.

We stratified participants into low and high groups based on their blood omega-3 index with the cutoff values of < 4% and > 5.5% respectively. Omega-3 indices approximately 4% and 5.5% correlate with relatively high and low risk for coronary heart disease, respectively [34]. Although there is no consensus in clinical practice on optimal omega-3 index values for cardiovascular health, there have been studies supporting the notion that a higher omega-3 index is cardioprotective [34, 35]. The American Heart Association recommends that patients with coronary heart diseases consume 1 g per day of EPA and DHA to lower the CVD risk. The recently approved highly purified prescription form of EPA (Icosapent ethyl) has been shown to significantly reduce cardiovascular risk in patients with hypertriglyceridemia [20], further promoting the use of omega-3 PUFA in CVD. We also reported beneficial modification of dietary omega-3 PUFA on the association between short-term exposure to ambient NO_2_ and respiratory and cardiovascular outcomes [36]. Taken together, these findings suggest that dietary omega-3 PUFA may confer cardioprotective benefits against adverse health effects of exposure to ambient air pollution in healthy adults even at levels below current air quality standards.

The findings of this study are noteworthy in several respects. First, this observational study was carried out with participants conducting their daily activities, making the findings more generalizable to real-life scenarios. Second, the 24-h dietary recall methodology employed to monitor dietary intake of EPA + DHA for each participant throughout the study indicated that the EPA + DHA intake levels remained relatively stable for both low and high omega-3 groups [37]. Third, this is the first study to report cardiovascular benefits of omega-3 PUFA against exposure to lower-than-NAAQS levels of PM_2.5_ and O_3_, indicating its potential as an interventional strategy against health effects of low-level air pollution. Fourth, relatively high omega-3 PUFA levels were achieved by participants through habitual fish and/or fish oil consumption, suggesting long-term cardiovascular benefits of dietary omega-3 PUFA against exposure to air pollution.

There are also a few limitations of this study. This study did not recruit participants who are considered susceptible to air pollution, such as the elderly and those with pre-existing cardiovascular diseases. Nonetheless, we have observed moderating effects of omega-3 PUFA on changes of cardiovascular biomarker associated with ambient air pollutants, and it is likely that these effects would be more prominent if susceptible population were included. This study employed a relatively small sample size and short-term exposure scenario; thus, caution is warranted inferring causal association and long-term implication of the findings. Only the health effects of PM_2.5_ and O_3_ were considered in the study while there might be other components of air pollution in play such as secondary organic aerosols. Furthermore, air pollution data were based on central air quality monitoring stations rather than individual exposure metrics such as location, time spent indoor vs. outdoor, and activity level, which could possibly introduce non-differential exposure misclassification. Finally, even though we have restricted dietary and medication usage during the study period, other factors such as lifestyle (exercise, balanced diet, stress, etc.) could be potential confounders.

## Conclusions

This observational study demonstrates that habitual dietary omega-3 PUFA may provide benefits in ameliorating the cardiovascular effects associated with short-term exposure to low levels of ambient air pollutants including PM_2.5_ and O_3_. These findings suggest that dietary omega-3 PUFA intake may offer a simple and effective interventional approach at an individual level to mitigate the adverse cardiovascular effects of exposure to ambient air pollution.

## Methods

### Study population and design

The study was carried out in Central North Carolina from October 2016 to September 2019. Healthy participants meeting the following criteria were recruited: 25–55 years old; body mass index (BMI) between 19 and 35; having no history of cardiovascular disease, chronic respiratory disease, cancer, uncontrolled hypertension (≥ 140 systolic, ≥ 90 diastolic), or diabetes; non-smokers for at least 1 year; not taking β-adrenergic receptor blockers, anti-inflammatory drugs, and statins. Participants were recruited from Research Triangle area in close proximity to the U.S. Environmental Protection Agency (U.S.EPA) Human Studies Facility (HSF) in North Carolina, USA.

Eligible participants were further screened and enrolled into low or high omega-3 PUFA groups meeting one of the following criteria: 1) As described previously [38], an inhouse open-ended dietary questionnaire was used to screen participants whose EPA + DHA intake was less than 0.5 g/week (low) or at least 3 g/week (high) for 6 months or longer; 2) Omega-3 index (OmegaQuant, Sioux Falls, SD), a measurement of EPA and DHA in erythrocyte membrane, was employed to screen participants whose omega-3 index was less than 4.0% (low) or at least 5.5% (high).

Each enrolled volunteer visited the Human Studies Facility (HSF) of the U.S.EPA for up to 5 sessions separated by at least 7 days between two sessions. Participants were instructed to keep their diet routine during the study and refrain from using any pain medications for two weeks before each session. Each session consists of two visits on consecutive days. On the first day, participants were outfitted with a Holter monitor and recorded continuously for 30 min. On the second day, venous blood was collected for biomarker measurements and Holter monitoring was recorded for 30 min. Written informed consent was given by all participants prior to enrollment. The study was approved by the Institutional Review Board of the University of North Carolina at Chapel Hill and the U.S.EPA and registered at ClinicalTrials.gov (NCT02921048).

### Exposure assessment

Hourly concentrations of ambient PM_2.5_ and O_3_ were obtained from a central air quality monitoring station (Millbrook) approximately 44 km (27 miles) from the HSF. Twenty four-hour average concentrations of PM_2.5_ were calculated from hourly pollutant data averaged between 9 and 8 AM, with a valid day defined as having at least 18 hourly measurements over the 24-h period. Daily maximum 8-h O_3_ concentrations were defined as the highest 8-h moving average concentrations between 9 and 8 AM. For missing data, an alternative central monitoring station (Durham Armory) approximately 18 km (11 miles) from the HSF was employed. Concentrations were assigned to each visit session (the day of blood sample collection is defined as lag0), as well as to 4 days prior (lag1–lag4), and the 5-day moving average (5dMA). Twenty four-hour averages of NO_2_ concentration, air temperature and relative humidity were collected from the same monitoring station.

### Venous blood samples

A portion of each blood sample was sent to a commercial lab (LabCorp, Burlington, NC) for quantification of blood lipids. The remainders of the blood samples were separated for plasma and stored at -80 ℃ prior to biomarker analysis. Commercially available multi-array plates were used to quantify levels of von Willebrand factor (vWF), tissue plasminogen activator (tPA), and D-dimer (MesoScale, Rockville, MD). In addition, vascular inflammation biomarkers including soluable intercellular adhesion molecule 1 (sICAM-1), soluable vascular cell adhesion molecule 1 (sVCAM-1), and serum amyloid A (SAA) were measured using a multiplex kit from MesoScale Diagnostics (Gaithersburg, MD). All experiments were performed per manufacturers’ instructions.

### Holter monitoring

As described previously [39], a Holter monitor was placed on the participants on both days of each session. The participants reclined in a dark room for 30 min and Holter were recorded using a H12 + 12-Lead ECG Recorder (Mortara, Milwaukee, WI). HRV and repolarization parameters were measured during the last 5 min of Holter recording. Time-domain measurements [standard deviation of normal-to-normal (SDNN), root-mean square of successive differences (rMSSD)] and frequency-domain measurements [very-low frequency (VLF), normalized low frequency (LFn), normalized high frequency (HFn), and low-to-high frequency power (LF/HF)] were measured. Cardiac repolarization was assessed by measuring the QT interval and corrected by heart rate (QTc). T wave complexity was measured in each beat by principal component analysis based on all 12 leads and averaged. QRS complexity and P wave complexity was calculated with the Mortara software.

### Statistical analysis

The data analysis was performed using R (version 3.6.2) with the “gamm4” package. To improve normality in the residuals, we log-transformed all dependent variables except for LFn and HFn. Generalized linear mixed models with random subject effects were employed to analyze the associations between exposure to air pollutants and cardiovascular biomarkers. Based on the repeated measurements in the same subjects, this approach assessed the within-subject variabilities in biomarkers under different exposure levels. The statistical model was adjusted for age, sex, race, BMI, long-term and seasonal trends, day of the week, temperature, and relative humidity. The long-term and seasonal trends were controlled for by a penalized spline of time with eight degrees of freedom (df) per year. Temperature (lag0-1 for high temperatures and lag 0–4 for low temperatures) and relative humidity (lag0-4) were incorporated as penalized splines with the df selected by the Generalized Cross Validation criterion. Linear terms of PM_2.5_ and O_3_ were included in the model separately to assess the immediate (lag0), delayed (lag1 to lag4), or cumulative (5-day moving average, 5dMA) effects. A product term of omega-3 groups and air pollutant concentrations was added to assess between-group differences. The results were interpreted as percent change from the mean of the measured outcome per interquartile range (IQR) increase of exposure. We also conducted two sensitivity analyses to test the robustness of the results. First, we restricted analyses to outcome data without outliers (defined as those lower than 1st quartile − 3 $\times$ IQR and those higher than 3rd quartile + 3 $\times$ IQR). Second, we adjusted the analyses using a 2-pollutant model; for example, we adjusted effect estimates of biomarkers per IQR increase in PM_2.5_ with either O_3_ or NO_2_ concentrations at the same lag. Statistical significance was set at a two-sided *p* < 0.05 for the air pollution effects and a two-sided *p* < 0.1 for the interaction with the two groups.

## Supplementary Information

**Additional file 1.** Omega-3 fatty acids attenuate cardiovascular effects of short-term exposure to ambient air pollution.

## Data Availability

The datasets generated and/or analyzed during the current study are available in the EPA ScienceHub repository.

## Abbreviations

CI: Confidence interval
DHA: Docosahexaenoic acid
EPA: Eicosapentaenoic acid
HDL: High-density lipoproteins
HFn: Normalized high frequency
HRV: Heart rate variability
IQR: Interquartile range
LDL: Low-density lipoproteins
LFn: Normalized low frequency
LF/HF: Low to high frequency power
PM_2.5_: Fine particulate matter
PUFA: Polyunsaturated fatty acids
QTc: Q-T corrected
RMSSD: Root mean square of successive differences
SAA: Serum amyloid A
SD: Standard deviation
SDNN: Standard deviation standard deviation of normal-to-normal
sICAM-1: Soluble intercellular adhesion molecule 1
SPM: Specialized pro-resolving mediator
sVCAM-1: Soluble vascular cell adhesion molecule 1
tPA: Tissue plasminogen activator
VLF: Very-low frequency
vWF: Von Willebrand factor
