# Supplementary material for: Omega-3 fatty acids attenuate cardiovascular effects of short-term exposure to ambient air pollution
Source: Part Fibre Toxicol. 2022 Feb 9;19:12. doi: 10.1186/s12989-022-00451-4 (PMC8826673; doi:10.1186/s12989-022-00451-4)
Supplement: Supplementary file 1 — Additional file 1. Omega-3 fatty acids attenuate cardiovascular effects of short-term exposure to ambient air pollution. [file 12989_2022_451_MOESM1_ESM.docx]

**Supplemental**

**Omega-3 Fatty Acids Attenuate Cardiovascular Effects of Short-term Exposure to Ambient Air Pollution**

Hao Chen, PhD ^a,*^; Siqi Zhang, PhD ^b,*^; Wan Shen, PhD ^a,c,*^; Claudia Salazar, BS ^d^, Alexandra Schneider, PhD ^b^; Lauren H. Wyatt, PhD ^d^; Ana G. Rappold, PhD ^d;^ David Diaz-Sanchez, PhD ^d^; Robert B. Devlin, PhD ^d^; James M. Samet, PhD, MPH ^d^; Haiyan Tong, MD, PhD ^d^

^a^ Oak Ridge Institute for Science Education, Oak Ridge, TN, USA; ^b^ Institute of Epidemiology, Helmholtz Zentrum München, Neuherberg, Germany; ^c^ Department of Public and Allied Health, Bowling Green State University, Bowling Green, OH, USA; ^d^ Public Health and Integrated Toxicology Division, Center for Public Health and Environmental Assessment, Office of Research and Development, U.S. Environmental Protection Agency, Chapel Hill, NC, USA.

^*^ These authors contribute equally to this work.

**Abbreviations**: CI, confidence interval; HDL, high-density lipoproteins; HFn, normalized high frequency; IQR, interquartile range; LDL, low-density lipoproteins; LFn, normalized low frequency; LF/HF, low to high frequency power; PM_2.5_, fine particulate matter; QTc, Q-T corrected; RMSSD, root mean square of successive differences; SAA, serum amyloid A; SD, standard deviation; SDNN, standard deviation standard deviation of normal-to-normal; sICAM-1, soluble intercellular adhesion molecule 1; sVCAM-1, soluble vascular cell adhesion molecule 1; tPA, tissue plasminogen activator; VLF, very-low frequency; vWF, von Willebrand factor.

**Supplemental Table 1**. Descriptive statistics of all biomarkers across all sessions.

| Outcome | All | | Low omega-3 group | | High omega-3 group | |
| --- | --- | --- | --- | --- | --- | --- |
|  | n | Mean (SD) | n | Mean (SD) | n | Mean (SD) |
| Blood lipids | | | | | | |
| Cholesterol (mg/dL) | 300 | 187.8 (36.1) | 137 | 185.2 (36.0) | 163 | 190 (36.2) |
| HDL (mg/dL) | 300 | 61 (16.4) | 137 | 57.8 (15.1) | 163 | 63.6 (16.9) ^*^ |
| LDL (mg/dL) | 300 | 106.7 (30.7) | 137 | 106.4 (33.6) | 163 | 107 (28.1) |
| Triglycerides (mg/dL) | 300 | 100.7 (55.2) | 137 | 105 (50.1) | 163 | 97.2 (59.1) |
| Vascular inflammation | | | | | | |
| SAA (pg/mL) | 301 | 3816 (5890) | 137 | 4089 (6928) | 164 | 3588 (4868) |
| sICAM-1 (pg/mL) | 301 | 244.6 (46.3) | 137 | 251.4 (54.3) | 164 | 239 (37.7) |
| sVCAM-1 (pg/mL) | 301 | 236.9 (52.3) | 137 | 237.9 (44) | 164 | 236.2 (58.4) |
| Coagulation / fibrinolysis | | | | | | |
| tPA (ng/mL) | 301 | 7.4 (5.1) | 137 | 7.3 (5.1) | 164 | 7.4 (5.2) |
| vWF (ng/mL) | 299 | 104.5 (42.8) | 136 | 101 (44.6) | 163 | 107.4 (41.2) |
| D-dimer (ng/mL) | 301 | 2002.2 (6658.7) | 137 | 2365.2 (9531.6) | 164 | 1698.9 (2366.4) |
| Heart rate variability and repolarization | | | | | | |
| SDNN (ms) | 294 | 69.6 (28.1) | 133 | 66.6 (21.3) | 161 | 72 (32.5) |
| RMSSD (ms) | 294 | 47.5 (30.8) | 133 | 44 (24.2) | 161 | 50.4 (35.1) |
| HFn | 294 | 38 (16.1) | 133 | 38.4 (16.6) | 161 | 37.7 (15.7) |
| LFn | 294 | 51.4 (15.9) | 133 | 50.6 (16.6) | 161 | 52 (15.3) |
| LF/HF | 294 | 1.9 (1.7) | 133 | 1.9 (1.7) | 161 | 1.9 (1.6) |
| VLF | 294 | 2095 (2480.3) | 133 | 1774.6 (1799.4) | 161 | 2359.6 (2904.9) |
| P Complexity (ms) | 294 | 140.9 (60) | 133 | 146.2 (68.9) | 161 | 136.5 (51.3) |
| QRS Complexity (ms) | 294 | 275.8 (135.3) | 133 | 283.7 (128.4) | 161 | 269.3 (140.9) |
| QTc (ms) | 294 | 411.8 (21.3) | 133 | 411.8 (20.7) | 161 | 411.9 (21.8) |
| T Complexity (ms) | 294 | 108.9 (63) | 133 | 114.8 (49.4) | 161 | 104 (72.1) |

**Supplemental Table 2**. Percent change (95% CI) in blood lipid markers per IQR increase in PM_2.5_ and O_3_ in the low and high omega-3 groups.

| Outcome | Lag (Day) | PM_2.5_ | | |  | O_3_ | | |
| --- | --- | --- | --- | --- | --- | --- | --- | --- |
|  |  | Low | High | *p*_interaction_ |  | Low | High | *p*_interaction_ |
| Total Cholesterol | Lag0 | 0.1 (-2.1, 2.3) | 1 (-1, 3) | 0.532 |  | 3.8 (0.9, 6.9) ^*^ | -0.2 (-2.7, 2.4) | 0.023 |
|  | Lag1 | -2.4 (-4.3, -0.4) ^*^ | -0.5 (-2.4, 1.5) | 0.146 |  | 0.6 (-2.3, 3.6) | -2.6 (-5.1, -0.1) ^*^ | 0.066 |
|  | Lag2 | -2.2 (-4, -0.4) ^*^ | -1.5 (-3.2, 0.2) | 0.550 |  | -3.5 (-6.2, -0.8) ^*^ | -1.7 (-4.2, 0.9) | 0.266 |
|  | Lag3 | -1.6 (-3.5, 0.3) | -0.2 (-1.9, 1.6) | 0.260 |  | -2.9 (-5.6, -0.2) ^*^ | -0.4 (-3.2, 2.5) | 0.148 |
|  | Lag4 | -1.5 (-3.4, 0.4) | -0.9 (-2.5, 0.8) | 0.610 |  | -2.9 (-5.2, -0.4) ^*^ | 0.2 (-2.2, 2.8) | 0.069 |
|  | 5dMA | -3.7 (-6.5, -0.8) ^*^ | -1.4 (-4, 1.3) | 0.211 |  | -4.2 (-8.9, 0.6) | -3.4 (-7.3, 0.7) | 0.716 |
| HDL | Lag0 | -1.1 (-4.2, 2.1) | 0.4 (-2.4, 3.4) | 0.467 |  | 0.7 (-3.4, 5) | 1.8 (-2, 5.7) | 0.679 |
|  | Lag1 | -2.4 (-5.2, 0.5) | -1.3 (-4, 1.6) | 0.564 |  | -3.9 (-7.9, 0.3) | -0.1 (-3.8, 3.7) | 0.135 |
|  | Lag2 | -0.9 (-3.7, 1.8) | -1.4 (-3.9, 1.2) | 0.810 |  | -5 (-8.8, -1) ^*^ | 0.6 (-3.2, 4.4) | 0.026 |
|  | Lag3 | -1.9 (-4.7, 0.9) | 0 (-2.5, 2.6) | 0.297 |  | -4.5 (-8.3, -0.6) ^*^ | 2.3 (-1.9, 6.6) | 0.009 |
|  | Lag4 | -0.3 (-3.1, 2.6) | -1.7 (-4, 0.8) | 0.461 |  | -3.6 (-7.1, -0.1) ^*^ | 1.2 (-2.4, 5) | 0.051 |
|  | 5dMA | -3.2 (-7.3, 1.1) | -2.1 (-5.9, 1.9) | 0.681 |  | -9.2 (-15.4, -2.4) ^*^ | 0.3 (-5.6, 6.5) | 0.007 |
| LDL | Lag0 | 0.3 (-2.9, 3.6) | 0 (-2.9, 3) | 0.885 |  | 4.2 (-0.1, 8.6) | -2.9 (-6.4, 0.9) | 0.006 |
|  | Lag1 | -2.7 (-5.5, 0.2) | -1.7 (-4.5, 1.1) | 0.637 |  | 1.1 (-3.1, 5.5) | -5.3 (-8.8, -1.8) ^*^ | 0.011 |
|  | Lag2 | -2.4 (-5, 0.3) | -1.7 (-4.1, 0.9) | 0.690 |  | -4.3 (-8.2, -0.2) | -2.1 (-5.8, 1.7) | 0.379 |
|  | Lag3 | -1.2 (-4, 1.6) | 0.9 (-1.6, 3.5) | 0.253 |  | -2.9 (-6.8, 1.1) | -0.3 (-4.4, 3.9) | 0.327 |
|  | Lag4 | -0.9 (-3.8, 2) | -0.6 (-3, 1.8) | 0.868 |  | -3.5 (-7, 0.1) | 1.2 (-2.5, 4.9) | 0.062 |
|  | 5dMA | -3.4 (-7.5, 0.9) | -1.7 (-5.6, 2.3) | 0.540 |  | -5.2 (-11.8, 1.9) | -5.7 (-11.3, 0.2) | 0.880 |
| Triglycerides | Lag0 | -1.5 (-8.7, 6.2) | 4.1 (-2.9, 11.5) | 0.261 |  | 3.1 (-6.7, 13.9) | 4 (-4.9, 13.9) | 0.877 |
|  | Lag1 | -0.8 (-7.4, 6.3) | 8.5 (1.4, 16) ^*^ | 0.055 |  | 5 (-5.2, 16.4) | 0.6 (-8, 10.1) | 0.488 |
|  | Lag2 | -3 (-9.3, 3.7) | -2.9 (-8.7, 3.2) | 0.984 |  | 3.1 (-6.8, 13.9) | -4.7 (-13, 4.4) | 0.202 |
|  | Lag3 | -0.6 (-7.1, 6.3) | -5.4 (-11, 0.5) | 0.266 |  | 0.7 (-8.6, 11) | -7 (-15.8, 2.8) | 0.209 |
|  | Lag4 | -4.6 (-10.9, 2.2) | -0.6 (-6.2, 5.4) | 0.359 |  | 3.1 (-5.5, 12.5) | -7.1 (-14.9, 1.3) | 0.079 |
|  | 5dMA | -5 (-14.4, 5.5) | -0.2 (-9.3, 9.9) | 0.457 |  | 6.6 (-10.3, 26.6) | -4.7 (-17.6, 10.1) | 0.196 |

Note: * *p* < 0.05 for significant association within a group.

**Supplemental Table 3**. Percent change (95% CI) in vascular inflammation markers per IQR increase in PM_2.5_ and O_3_ in the low and high omega-3 groups.

| Outcome | Lag (Day) | PM_2.5_ | | |  | O_3_ | | |  |
| --- | --- | --- | --- | --- | --- | --- | --- | --- | --- |
|  |  | Low | High | *p*_interaction_ |  | Low | High | *p*_interaction_ |  |
| SAA | Lag0 | 3.1 (-11.9, 20.6) | 3.3 (-10.6, 19.3) | 0.985 |  | 9.9 (-10.6, 35.2) | 0.7 (-16.5, 21.4) | 0.485 |  |
|  | Lag1 | 9.5 (-5.3, 26.7) | -3.3 (-15.9, 11.1) | 0.202 |  | 27.2 (3.1, 57) ^*^ | 4.1 (-13.3, 25) | 0.114 |  |
|  | Lag2 | -0.9 (-13.5, 13.7) | 0.1 (-11.5, 13.3) | 0.911 |  | 17.1 (-5, 44.4) | -3.7 (-20.2, 16.3) | 0.123 |  |
|  | Lag3 | 5.9 (-8.1, 21.9) | -5.7 (-17.1, 7.2) | 0.215 |  | 13.3 (-7.8, 39.3) | 9.6 (-11.3, 35.5) | 0.800 |  |
|  | Lag4 | 9.9 (-4.9, 27) | 1.9 (-9.7, 15) | 0.417 |  | 0.4 (-16.4, 20.5) | 7.6 (-9.9, 28.5) | 0.570 |  |
|  | 5dMA | 12.2 (-9.6, 39.4) | -3 (-20.7, 18.7) | 0.290 |  | 47.5 (2.3, 112.7) ^*^ | 18.9 (-12.7, 61.9) | 0.230 |  |
| sICAM-1 | Lag0 | 0.4 (-2.2, 3) | 1 (-1.4, 3.4) | 0.729 |  | 4.4 (1, 7.9) ^*^ | 0 (-3, 3) | 0.034 |  |
|  | Lag1 | 0.6 (-1.8, 3) | 2.1 (-0.2, 4.5) | 0.342 |  | 2.3 (-1.2, 6) | 0.2 (-2.8, 3.3) | 0.316 |  |
|  | Lag2 | -1.5 (-3.7, 0.8) | 0.2 (-1.9, 2.3) | 0.276 |  | 0.6 (-2.8, 4.2) | 0.7 (-2.4, 3.9) | 0.980 |  |
|  | Lag3 | 0.3 (-2, 2.7) | -0.4 (-2.5, 1.8) | 0.681 |  | -1.5 (-4.7, 1.9) | -2.8 (-6.1, 0.6) | 0.533 |  |
|  | Lag4 | 0 (-2.4, 2.4) | -0.7 (-2.7, 1.3) | 0.642 |  | -0.7 (-3.6, 2.4) | -3 (-5.8, -0.1) ^*^ | 0.246 |  |
|  | 5dMA | -0.2 (-3.7, 3.5) | 0.7 (-2.6, 4.1) | 0.708 |  | 1.9 (-4.1, 8.3) | -2.3 (-7.2, 2.8) | 0.156 |  |
| sVCAM-1 | Lag0 | 0.8 (-2, 3.6) | 0.9 (-1.5, 3.5) | 0.923 |  | 2.5 (-1.2, 6.4) | -2.1 (-5.3, 1.2) | 0.042 |  |
|  | Lag1 | 1 (-1.6, 3.6) | 1.4 (-1, 4) | 0.787 |  | 2.1 (-1.7, 6.1) | -0.3 (-3.5, 3) | 0.285 |  |
|  | Lag2 | 0.4 (-2, 2.9) | -0.5 (-2.7, 1.7) | 0.556 |  | 0.8 (-2.8, 4.6) | -0.8 (-4.1, 2.5) | 0.468 |  |
|  | Lag3 | 2 (-0.5, 4.5) | -0.8 (-3, 1.5) | 0.100 |  | -1.9 (-5.3, 1.7) | -3.2 (-6.7, 0.4) | 0.554 |  |
|  | Lag4 | 1.3 (-1.3, 3.9) | -1 (-3.1, 1.1) | 0.167 |  | -1 (-4.1, 2.3) | -1.9 (-4.9, 1.2) | 0.676 |  |
|  | 5dMA | 2.5 (-1.4, 6.5) | -0.1 (-3.5, 3.5) | 0.302 |  | 0 (-6.1, 6.5) | -3.7 (-8.7, 1.5) | 0.241 |  |

* *p* < 0.05 for significant association within a group.

**Supplemental Table 4**. Percent change (95% CI) in coagulation and fibrinolysis markers per IQR increase in PM_2.5_ and O_3_ in the low and high omega-3 groups.

| Outcome | Lag (Day) | PM_2.5_ | | |  | O_3_ | | |
| --- | --- | --- | --- | --- | --- | --- | --- | --- |
|  |  | Low | High | *p*_interaction_ |  | Low | High | *p*_interaction_ |
| tPA | Lag0 | -1.3 (-6.6, 4.3) | 1.4 (-3.5, 6.6) | 0.438 |  | 0.9 (-6.2, 8.4) | 3.1 (-3.4, 10.1) | 0.616 |
|  | Lag1 | -2.6 (-7.5, 2.5) | 0.2 (-4.6, 5.2) | 0.409 |  | 0.9 (-6.4, 8.9) | -0.7 (-6.9, 6) | 0.724 |
|  | Lag2 | 2.7 (-2.1, 7.7) | -1.5 (-5.6, 2.9) | 0.189 |  | -1.7 (-8.7, 5.7) | 1.8 (-4.7, 8.8) | 0.430 |
|  | Lag3 | 6.1 (1, 11.4) ^*^ | 0.8 (-3.5, 5.3) | 0.116 |  | 2.5 (-4.5, 10) | 6.3 (-1.1, 14.2) | 0.429 |
|  | Lag4 | -2.8 (-7.6, 2.2) | 2.3 (-1.9, 6.7) | 0.119 |  | -0.8 (-7, 5.8) | 3 (-3.2, 9.6) | 0.387 |
|  | 5dMA | 1.1 (-6.3, 9.2) | 1.7 (-5.1, 9) | 0.903 |  | 2.6 (-9.6, 16.4) | 7 (-3.7, 19) | 0.513 |
| vWF | Lag0 | 6.3 (1.1, 11.8) ^*^ | 0.8 (-3.6, 5.4) | 0.099 |  | 14 (6.9, 21.5) ^*^ | -4 (-9.3, 1.7) | 0.000 |
|  | Lag1 | 1.4 (-3.2, 6.3) | 0.9 (-3.5, 5.5) | 0.875 |  | 6.4 (-0.6, 13.8) | -5.3 (-10.6, 0.4) | 0.005 |
|  | Lag2 | -4.1 (-8.2, 0.2) | -2.2 (-5.9, 1.8) | 0.486 |  | -1.8 (-8.2, 5) | -5.2 (-10.7, 0.6) | 0.393 |
|  | Lag3 | -5.8 (-10, -1.5) ^*^ | -0.5 (-4.4, 3.5) | 0.067 |  | -0.7 (-7, 6.1) | -6.3 (-12.3, 0.1) | 0.171 |
|  | Lag4 | -4.9 (-9.2, -0.4) ^*^ | -0.7 (-4.4, 3.2) | 0.148 |  | -1 (-6.8, 5.1) | -0.8 (-6.4, 5.1) | 0.964 |
|  | 5dMA | -4.7 (-11.1, 2.2) | -1.9 (-7.9, 4.5) | 0.518 |  | 6.3 (-5.3, 19.2) | -8.9 (-17.2, 0.3) | 0.009 |
| D-dimer | Lag0 | 5.6 (-6.7, 19.5) | -1.9 (-12.4, 9.8) | 0.358 |  | 15 (-2.3, 35.3) | 8.4 (-6.5, 25.6) | 0.547 |
|  | Lag1 | 13.1 (1, 26.8) ^*^ | -0.6 (-11, 10.9) | 0.088 |  | 17.1 (-0.9, 38.4) | 4.7 (-9.4, 21.1) | 0.261 |
|  | Lag2 | 2.9 (-7.8, 14.9) | -4.4 (-13.5, 5.6) | 0.311 |  | 9.2 (-7.5, 28.8) | 0.6 (-13.3, 16.7) | 0.413 |
|  | Lag3 | -6 (-15.9, 5.1) | -5.5 (-14.5, 4.4) | 0.943 |  | 9.2 (-7, 28.2) | 2.1 (-12.9, 19.7) | 0.505 |
|  | Lag4 | -11.1 (-20.6, -0.6) ^*^ | -7.4 (-15.7, 1.8) | 0.570 |  | -5.3 (-17.9, 9.2) | 1.6 (-11.4, 16.6) | 0.455 |
|  | 5dMA | -0.1 (-15.8, 18.5) | -10 (-23.1, 5.4) | 0.336 |  | 27.9 (-3.3, 69.2) | 13.4 (-10.4, 43.4) | 0.381 |

* *p* < 0.05 for significant association within a group.

**Supplemental Table 5**. Percent change (95% CI) in HRV markers per IQR increase in PM_2.5_ and O_3_ in the low and high omega-3 groups.

| Outcome | Lag (Day) | PM_2.5_ | | |  | O_3_ | | |
| --- | --- | --- | --- | --- | --- | --- | --- | --- |
|  |  | Low | High | *p*_interaction_ |  | Low | High | *p*_interaction_ |
| SDNN | Lag0 | -1.4 (-6.2, 3.7) | -1.6 (-6, 3) | 0.945 |  | 0.4 (-6.1, 7.4) | 0.9 (-4.9, 7.2) | 0.891 |
|  | Lag1 | -1 (-5.6, 3.8) | -2 (-6.2, 2.5) | 0.770 |  | 1.7 (-5.2, 9) | -0.5 (-6.2, 5.6) | 0.607 |
|  | Lag2 | 3.7 (-0.9, 8.6) | -0.5 (-4.4, 3.6) | 0.163 |  | 1.7 (-5.1, 8.9) | -1.4 (-7.1, 4.8) | 0.469 |
|  | Lag3 | 3.1 (-1.5, 7.9) | 0 (-4, 4.2) | 0.311 |  | 2 (-4.4, 8.9) | -1.3 (-7.5, 5.4) | 0.433 |
|  | Lag4 | 2.2 (-2.4, 7) | -0.1 (-3.9, 3.8) | 0.440 |  | 2.6 (-3.2, 8.9) | 4.2 (-1.5, 10.3) | 0.704 |
|  | 5dMA | 3.3 (-3.8, 10.8) | -1.3 (-7.4, 5.2) | 0.308 |  | 6.5 (-5.3, 19.7) | 2.7 (-6.7, 13.1) | 0.549 |
| RMSSD | Lag0 | -3.8 (-10.8, 3.8) | -1.9 (-8.3, 5.1) | 0.682 |  | -1.8 (-11.3, 8.7) | 0 (-8.6, 9.5) | 0.765 |
|  | Lag1 | -1.5 (-8.3, 5.8) | -1 (-7.5, 5.9) | 0.918 |  | 4.7 (-5.7, 16.3) | 1.7 (-6.9, 11.2) | 0.646 |
|  | Lag2 | 3.8 (-3, 11.2) | 0.3 (-5.6, 6.5) | 0.429 |  | 1.7 (-8.2, 12.8) | 0.2 (-8.5, 9.8) | 0.815 |
|  | Lag3 | 2.9 (-3.9, 10.1) | 0.2 (-5.8, 6.5) | 0.557 |  | 4.3 (-5.4, 14.9) | 0.7 (-8.8, 11.1) | 0.581 |
|  | Lag4 | 1.3 (-5.5, 8.6) | 0.3 (-5.4, 6.3) | 0.815 |  | 3.1 (-5.7, 12.6) | 7.3 (-1.5, 16.8) | 0.503 |
|  | 5dMA | 1.5 (-8.7, 12.9) | -0.4 (-9.5, 9.6) | 0.768 |  | 10.2 (-7.6, 31.5) | 7.2 (-7.2, 23.9) | 0.757 |
| HFn | Lag0 | -3 (-10, 4) | -4.4 (-10.8, 1.9) | 0.755 |  | 0 (-9.5, 9.6) | 0.5 (-8, 9.1) | 0.929 |
|  | Lag1 | -1.2 (-7.8, 5.4) | -3 (-9.3, 3.3) | 0.684 |  | 2.6 (-7.2, 12.4) | -0.3 (-8.6, 8) | 0.620 |
|  | Lag2 | 0.1 (-6.4, 6.5) | -2.8 (-8.5, 2.8) | 0.489 |  | -3.8 (-13.4, 5.8) | -0.4 (-8.9, 8) | 0.567 |
|  | Lag3 | 0.7 (-5.6, 7.1) | -2 (-7.8, 3.7) | 0.520 |  | -0.4 (-9.5, 8.6) | 5.9 (-3.2, 15) | 0.282 |
|  | Lag4 | 0.3 (-6.2, 6.7) | 1.2 (-4.2, 6.6) | 0.829 |  | -0.2 (-8.5, 8) | 5.2 (-2.6, 13.1) | 0.323 |
|  | 5dMA | -1.6 (-11.5, 8.2) | -5 (-13.9, 3.9) | 0.588 |  | 0.9 (-15.5, 17.4) | 4.5 (-9, 17.9) | 0.669 |
| LFn | Lag0 | 3.2 (-2.4, 8.9) | 5.1 (0, 10.3) | 0.608 |  | -0.4 (-8.2, 7.3) | -1.3 (-8.2, 5.6) | 0.850 |
|  | Lag1 | 0.3 (-5.1, 5.6) | 3.5 (-1.6, 8.5) | 0.368 |  | -3.4 (-11.2, 4.5) | -0.4 (-7, 6.3) | 0.521 |
|  | Lag2 | 1.3 (-3.9, 6.5) | 1.5 (-3.1, 6.1) | 0.947 |  | 0.4 (-7.4, 8.1) | 0.4 (-6.5, 7.2) | 1.000 |
|  | Lag3 | 0.6 (-4.5, 5.8) | -0.8 (-5.5, 3.9) | 0.673 |  | 0.8 (-6.6, 8.2) | -4.2 (-11.6, 3.2) | 0.297 |
|  | Lag4 | 0.7 (-4.5, 5.9) | -2.8 (-7.2, 1.6) | 0.300 |  | -0.5 (-7.1, 6.2) | -5.4 (-11.8, 0.9) | 0.263 |
|  | 5dMA | 2.7 (-5.3, 10.8) | 2.8 (-4.5, 10) | 0.996 |  | -3.7 (-17, 9.6) | -5.1 (-16, 5.8) | 0.825 |
| LF/HF | Lag0 | 7.4 (-6.1, 22.9) | 12.2 (-0.7, 26.8) | 0.619 |  | 0.3 (-16.5, 20.6) | -1.8 (-16.7, 15.7) | 0.842 |
|  | Lag1 | 2 (-10.3, 15.9) | 8 (-4.3, 21.8) | 0.499 |  | -8 (-23.8, 11.2) | 0.6 (-14.3, 18.1) | 0.427 |
|  | Lag2 | 2.3 (-9.6, 15.9) | 5.3 (-5.6, 17.5) | 0.720 |  | 5 (-12.8, 26.5) | 1 (-14.2, 19) | 0.729 |
|  | Lag3 | 0 (-11.5, 13.1) | 1.8 (-8.9, 13.8) | 0.826 |  | 1.5 (-14.8, 20.9) | -10.5 (-25, 6.7) | 0.264 |
|  | Lag4 | 1.4 (-10.5, 14.8) | -4.1 (-13.6, 6.4) | 0.492 |  | -0.8 (-15.3, 16.3) | -11.1 (-23.6, 3.4) | 0.301 |
|  | 5dMA | 6.2 (-12.2, 28.5) | 10.3 (-7.1, 31.1) | 0.748 |  | -5.4 (-31.1, 30) | -9.6 (-30.3, 17.2) | 0.772 |
| VLF | Lag0 | -20.2 (-34.8, -2.2) ^*^ | -11.1 (-26.1, 6.9) | 0.415 |  | -20.8 (-40.1, 4.7) | -2.5 (-23.9, 24.9) | 0.204 |
|  | Lag1 | -6.3 (-22.7, 13.7) | -9.7 (-24.9, 8.5) | 0.769 |  | 8.8 (-18.1, 44.4) | 8.2 (-14.8, 37.5) | 0.976 |
|  | Lag2 | 0.4 (-16.8, 21.2) | -10.1 (-23.9, 6.3) | 0.372 |  | 20.1 (-9.2, 58.7) | 7.5 (-15.8, 37.3) | 0.512 |
|  | Lag3 | 12 (-7.1, 34.9) | 10.1 (-7, 30.2) | 0.890 |  | 12.3 (-13.7, 46.2) | -9.2 (-30.2, 18) | 0.204 |
|  | Lag4 | -0.2 (-17.4, 20.6) | 0.3 (-14.5, 17.8) | 0.966 |  | -3.6 (-24.1, 22.5) | -3.3 (-23.1, 21.7) | 0.984 |
|  | 5dMA | -6.2 (-29.6, 25.1) | -8.7 (-29.8, 18.8) | 0.881 |  | 6.5 (-33.9, 71.6) | 0.8 (-31.9, 49.1) | 0.816 |

Note: * *p* < 0.05 for significant association within a group.

**Supplemental Table 6**. Percent change (95% CI) in repolarization markers per IQR increase in PM_2.5_ and O_3_ in the low and high omega-3 groups.

| Outcome | Lag (Day) | PM_2.5_ | | |  | O_3_ | | |
| --- | --- | --- | --- | --- | --- | --- | --- | --- |
|  |  | Low | High | *p*_interaction_ |  | Low | High | *p*_interaction_ |
| P Complexity | Lag0 | 2.1 (-4.8, 9.6) | 2.8 (-3.6, 9.6) | 0.887 |  | 1 (-8.4, 11.3) | 1.2 (-7.2, 10.2) | 0.976 |
|  | Lag1 | 0.5 (-5.9, 7.3) | 4.9 (-1.5, 11.8) | 0.323 |  | 0.7 (-8.6, 11) | 0 (-7.9, 8.5) | 0.898 |
|  | Lag2 | 2.4 (-3.9, 9.1) | 5.5 (-0.3, 11.6) | 0.476 |  | -4.9 (-13.5, 4.5) | -4 (-11.7, 4.4) | 0.867 |
|  | Lag3 | 2.5 (-3.9, 9.2) | 2.1 (-3.5, 8.1) | 0.940 |  | -4.2 (-12.7, 5) | -1.2 (-10, 8.4) | 0.602 |
|  | Lag4 | -1.5 (-7.6, 4.9) | 7.7 (2.1, 13.6) ^*^ | 0.031 |  | -2.5 (-10.3, 5.8) | 7.2 (-1, 16) | 0.086 |
|  | 5dMA | 3.4 (-6.2, 14) | 12 (2.5, 22.5) ^*^ | 0.187 |  | -5.6 (-19.9, 11.4) | 0.3 (-12.3, 14.8) | 0.471 |
| QRS Complexity | Lag0 | 2.4 (-3.5, 8.6) | 4.7 (-0.7, 10.4) | 0.563 |  | 2.4 (-5.6, 11.1) | 1.1 (-6, 8.7) | 0.793 |
|  | Lag1 | -0.5 (-6, 5.3) | 0.4 (-4.8, 5.9) | 0.819 |  | -5.6 (-13.1, 2.5) | -3.4 (-9.9, 3.6) | 0.637 |
|  | Lag2 | -1.5 (-6.6, 3.9) | 0.9 (-3.7, 5.8) | 0.485 |  | -6.6 (-13.8, 1.3) | -2.4 (-9.1, 4.8) | 0.378 |
|  | Lag3 | -0.9 (-6, 4.4) | 1 (-3.7, 6) | 0.572 |  | -9 (-15.7, -1.8) | -3.8 (-11, 3.9) | 0.268 |
|  | Lag4 | 1.9 (-3.5, 7.7) | 0.3 (-4.1, 5) | 0.662 |  | 1.6 (-5.4, 9) | 2.5 (-4.2, 9.7) | 0.851 |
|  | 5dMA | 0 (-8.1, 8.8) | 3 (-4.5, 11.1) | 0.569 |  | -10.9 (-22.5, 2.5) | -4.6 (-14.9, 6.9) | 0.339 |
| QTc | Lag0 | 0.4 (-0.2, 1) | 0.5 (-0.1, 1) | 0.912 |  | 0.6 (-0.2, 1.4) | 0.3 (-0.4, 1.1) | 0.558 |
|  | Lag1 | 0.4 (-0.2, 1) | 0.2 (-0.3, 0.8) | 0.649 |  | 0.6 (-0.3, 1.5) | -0.1 (-0.8, 0.6) | 0.158 |
|  | Lag2 | 0.2 (-0.4, 0.7) | 0.1 (-0.4, 0.6) | 0.883 |  | 0.2 (-0.7, 1) | 0 (-0.7, 0.8) | 0.750 |
|  | Lag3 | 0.3 (-0.2, 0.9) | 0.3 (-0.2, 0.8) | 0.892 |  | 0.4 (-0.4, 1.2) | -0.2 (-1, 0.6) | 0.284 |
|  | Lag4 | 0.3 (-0.2, 0.9) | -0.2 (-0.7, 0.2) | 0.122 |  | 0.4 (-0.4, 1.1) | -0.2 (-0.9, 0.5) | 0.253 |
|  | 5dMA | 0.8 (-0.1, 1.6) | 0.4 (-0.3, 1.2) | 0.536 |  | 1.3 (-0.1, 2.8) | 0.2 (-1, 1.4) | 0.127 |
| T Complexity | Lag0 | 2.9 (-3.4, 9.8) | 0.7 (-5, 6.7) | 0.591 |  | -2.6 (-10.8, 6.3) | 5 (-2.9, 13.5) | 0.155 |
|  | Lag1 | 5.1 (-1.1, 11.7) | 2.3 (-3.4, 8.3) | 0.501 |  | -6.1 (-14.2, 2.8) | 2.7 (-4.9, 10.8) | 0.099 |
|  | Lag2 | 3.5 (-2.4, 9.8) | -3.1 (-8, 2.1) | 0.085 |  | -2.3 (-10.6, 6.7) | 0.5 (-7.1, 8.7) | 0.604 |
|  | Lag3 | 3.5 (-2.4, 9.6) | -1.3 (-6.4, 4) | 0.220 |  | -2.2 (-10, 6.4) | 0.1 (-8, 9) | 0.666 |
|  | Lag4 | -1 (-6.7, 5.1) | -1.7 (-6.5, 3.3) | 0.852 |  | -2.1 (-9.3, 5.7) | 4.5 (-2.9, 12.4) | 0.211 |
|  | 5dMA | 5.7 (-3.5, 15.7) | -1.2 (-9, 7.3) | 0.241 |  | -7 (-20.1, 8.3) | 4.6 (-7.7, 18.4) | 0.131 |

Note: * *p* < 0.05 for significant association within a group.

**Supplemental Table 7**. Percent change (95% CI) in biomarkers per IQR increase in one air pollutant adjusted for the other pollutant of the same lag in the low and high omega-3 groups.

| Outcome | Lag (Day) | Effects of PM_2.5_ adjust for O_3_ | | | Effects of O_3_ adjust for PM_2.5_ | | |
| --- | --- | --- | --- | --- | --- | --- | --- |
|  |  | Low | High | *p*_interaction_ | Low | High | *p*_interaction_ |
| Total cholesterol | Lag0 | 0.1 (-2.1, 2.4) | 0.5 (-1.8, 2.8) | 0.797 | 3.7 (0.6, 6.8) * | -0.5 (-3.2, 2.3) | 0.021 |
|  | Lag1 | -2.1 (-4.1, -0.1) * | -0.2 (-2.2, 1.8) | 0.145 | 1 (-2, 4.1) | -2.1 (-4.7, 0.5) | 0.078 |
|  | Lag2 | -1.8 (-3.7, 0.2) | -1.1 (-2.9, 0.7) | 0.599 | -2.8 (-5.6, 0.2) | -0.7 (-3.3, 2) | 0.229 |
|  | Lag3 | -1.3 (-3.2, 0.7) | 0 (-1.7, 1.8) | 0.297 | -2.7 (-5.4, 0) * | 0 (-2.9, 3.1) | 0.123 |
|  | Lag4 | -1.2 (-3.3, 0.9) | -0.6 (-2.4, 1.2) | 0.636 | -2.2 (-4.8, 0.4) | 0.9 (-1.8, 3.7) | 0.064 |
|  | 5dMA | -3.2 (-6.1, -0.3) * | -1 (-3.7, 1.8) | 0.214 | -3.2 (-8, 1.8) | -2.3 (-6.4, 2) | 0.703 |
| HDL | Lag0 | -1.1 (-4.2, 2.2) | 0.3 (-3, 3.6) | 0.531 | 1 (-3.3, 5.5) | 1.7 (-2.3, 5.9) | 0.767 |
|  | Lag1 | -2.1 (-5, 0.9) | -1 (-3.9, 2) | 0.574 | -3.3 (-7.4, 1) | 0.7 (-3.1, 4.7) | 0.121 |
|  | Lag2 | -0.4 (-3.3, 2.5) | -0.7 (-3.4, 2.1) | 0.894 | -4.7 (-8.8, -0.4) * | 1 (-2.9, 5.1) | 0.025 |
|  | Lag3 | -1.8 (-4.6, 1.2) | 0.1 (-2.5, 2.8) | 0.318 | -4.2 (-8.1, -0.2) * | 2.9 (-1.5, 7.4) | 0.008 |
|  | Lag4 | 0 (-3.1, 3.2) | -1.5 (-4, 1.2) | 0.447 | -3 (-6.8, 0.9) | 1.9 (-2.1, 6) | 0.050 |
|  | 5dMA | -2.8 (-7.1, 1.6) | -1.7 (-5.7, 2.4) | 0.685 | -8.1 (-14.6, -1.1) * | 1.5 (-4.6, 8.1) | 0.007 |
| LDL | Lag0 | 0.5 (-2.8, 3.9) | 0 (-3.2, 3.4) | 0.835 | 4 (-0.4, 8.6) | -3.1 (-6.9, 0.8) | 0.006 |
|  | Lag1 | -2.2 (-5.1, 0.8) | -1.2 (-4.1, 1.8) | 0.626 | 1.7 (-2.6, 6.2) | -4.6 (-8.2, -0.8) * | 0.014 |
|  | Lag2 | -1.8 (-4.6, 1.2) | -1 (-3.7, 1.7) | 0.7 | -3.6 (-7.7, 0.8) | -1 (-4.9, 3) | 0.316 |
|  | Lag3 | -0.8 (-3.7, 2.1) | 1.2 (-1.4, 3.9) | 0.283 | -3 (-7, 1.1) | -0.5 (-4.8, 4) | 0.331 |
|  | Lag4 | -0.6 (-3.7, 2.6) | -0.4 (-3, 2.3) | 0.895 | -3.2 (-6.9, 0.7) | 1.5 (-2.4, 5.7) | 0.061 |
|  | 5dMA | -2.6 (-6.8, 1.9) | -0.9 (-4.9, 3.2) | 0.545 | -4.4 (-11.2, 3) | -4.9 (-10.7, 1.4) | 0.886 |
| Triglycerides | Lag0 | -2.4 (-9.7, 5.5) | 2.4 (-5.3, 10.8) | 0.349 | 3.1 (-7, 14.3) | 3.7 (-5.8, 14.1) | 0.933 |
|  | Lag1 | -1 (-7.8, 6.4) | 8.5 (1.1, 16.4) * | 0.054 | 3.4 (-6.9, 14.7) | -1.1 (-9.9, 8.6) | 0.474 |
|  | Lag2 | -3.1 (-9.7, 4.1) | -3 (-9.2, 3.7) | 0.982 | 4.7 (-5.9, 16.5) | -3.6 (-12.4, 6.2) | 0.187 |
|  | Lag3 | -0.2 (-6.9, 7.1) | -5.1 (-10.9, 0.9) | 0.255 | 1.7 (-7.9, 12.3) | -5.4 (-14.7, 4.9) | 0.255 |
|  | Lag4 | -4.4 (-11.3, 3.1) | -0.4 (-6.4, 6.1) | 0.365 | 4.6 (-4.9, 15) | -5.8 (-14.3, 3.5) | 0.079 |
|  | 5dMA | -5 (-14.7, 5.7) | -0.2 (-9.6, 10.2) | 0.457 | 8 (-9.6, 29) | -3.5 (-17.1, 12.2) | 0.195 |
| tPA | Lag0 | -2.4 (-7.7, 3.3) | 2.5 (-3, 8.4) | 0.183 | 1 (-6.3, 8.8) | 3.4 (-3.6, 10.8) | 0.601 |
|  | Lag1 | -2.6 (-7.6, 2.7) | 0.2 (-4.8, 5.4) | 0.416 | 1.5 (-6, 9.6) | -0.2 (-6.8, 6.9) | 0.717 |
|  | Lag2 | 2.9 (-2.2, 8.3) | -1 (-5.5, 3.7) | 0.229 | -2.8 (-10, 4.9) | 1.6 (-5.2, 8.9) | 0.321 |
|  | Lag3 | 5.5 (0.3, 11) * | 0.4 (-4, 5) | 0.13 | 1.2 (-5.8, 8.8) | 4.6 (-3, 12.8) | 0.476 |
|  | Lag4 | -3.4 (-8.5, 2) | 1.8 (-2.8, 6.5) | 0.113 | -0.6 (-7.3, 6.6) | 3.3 (-3.6, 10.6) | 0.387 |
|  | 5dMA | 0.2 (-7.4, 8.4) | 0.9 (-6.1, 8.4) | 0.888 | 2.3 (-10.2, 16.5) | 6.7 (-4.4, 19.1) | 0.515 |
| vWF | Lag0 | 6.6 (1.3, 12.2) * | -1.4 (-6.2, 3.7) | 0.02 | 13.2 (6.1, 20.8) * | -5.7 (-11.2, 0.1) | 0.000 |
|  | Lag1 | 1.8 (-3, 6.7) | 1.5 (-3.1, 6.2) | 0.926 | 5.9 (-1.1, 13.4) | -6.3 (-11.7, -0.4) * | 0.003 |
|  | Lag2 | -3.4 (-7.8, 1.1) | -1.7 (-5.8, 2.6) | 0.539 | -0.7 (-7.4, 6.6) | -4.2 (-10, 2) | 0.382 |
|  | Lag3 | -5.4 (-9.7, -0.9) * | -0.2 (-4.2, 4) | 0.075 | -0.1 (-6.6, 6.7) | -5 (-11.4, 1.7) | 0.240 |
|  | Lag4 | -5.4 (-10, -0.5) * | -1.1 (-5.1, 3.1) | 0.143 | 0.9 (-5.4, 7.6) | 1.2 (-4.9, 7.8) | 0.931 |
|  | 5dMA | -4.2 (-10.9, 2.9) | -1.5 (-7.7, 5.1) | 0.525 | 7.7 (-4.3, 21.2) | -7.6 (-16.3, 2.1) | 0.010 |
| D-dimer | Lag0 | 3.6 (-8.7, 17.6) | -7.3 (-18.3, 5.1) | 0.18 | 16.4 (-1.7, 37.8) | 8.8 (-6.9, 27.3) | 0.501 |
|  | Lag1 | 11.5 (-0.9, 25.4) | -2.1 (-12.7, 9.7) | 0.09 | 15.4 (-2.7, 36.9) | 2.3 (-12.1, 19) | 0.225 |
|  | Lag2 | 1.2 (-9.9, 13.8) | -5.7 (-15.3, 5) | 0.343 | 12.9 (-5.2, 34.4) | 1.9 (-12.9, 19.1) | 0.309 |
|  | Lag3 | -7.9 (-17.9, 3.2) | -6.9 (-15.9, 3) | 0.884 | 13.6 (-3.4, 33.4) | 6.8 (-9.3, 25.7) | 0.541 |
|  | Lag4 | -13.4 (-23.3, -2.2) * | -9.4 (-18.3, 0.4) | 0.539 | 2.8 (-11.8, 19.8) | 10.3 (-4.9, 28) | 0.449 |
|  | 5dMA | -3.9 (-19.3, 14.4) | -13.2 (-26.1, 2) | 0.348 | 34.4 (0.9, 79) * | 19.1 (-6.6, 51.9) | 0.380 |
|  |  |  |  |  |  |  |  |
| Supplemental Table 7 continued | | | | | | | |
| SAA | Lag0 | 5.3 (-10.4, 23.7) | 4.5 (-10.9, 22.5) | 0.943 | 6.3 (-14.1, 31.6) | -2.7 (-20.2, 18.6) | 0.485 |
|  | Lag1 | 8.2 (-6.8, 25.6) | -6.3 (-18.9, 8.2) | 0.138 | 25.7 (1.2, 56) * | 1.9 (-15.9, 23.5) | 0.102 |
|  | Lag2 | -2.1 (-15.3, 13.1) | 1.7 (-11, 16.2) | 0.678 | 16.7 (-6.1, 45.1) | -5 (-21.9, 15.4) | 0.104 |
|  | Lag3 | 3 (-11, 19.2) | -8.3 (-19.5, 4.4) | 0.219 | 11.4 (-9.5, 37.1) | 6.3 (-14.2, 31.7) | 0.726 |
|  | Lag4 | 8.2 (-7.5, 26.5) | 0.7 (-11.7, 14.8) | 0.447 | -3.2 (-20.6, 18.1) | 2.6 (-15.4, 24.5) | 0.637 |
|  | 5dMA | 8 (-13.6, 35) | -8.4 (-25.2, 12.4) | 0.232 | 36.2 (-5.8, 97) | 10.1 (-19.5, 50.5) | 0.241 |
| sICAM-1 | Lag0 | 0.6 (-2, 3.3) | 0.4 (-2.2, 3.1) | 0.925 | 4.1 (0.6, 7.7) * | -0.5 (-3.6, 2.7) | 0.029 |
|  | Lag1 | 0.4 (-2.1, 2.9) | 2 (-0.4, 4.5) | 0.313 | 1.8 (-1.7, 5.5) | -0.4 (-3.5, 2.8) | 0.290 |
|  | Lag2 | -1.9 (-4.2, 0.5) | -0.4 (-2.6, 1.8) | 0.33 | 1.3 (-2.3, 5.1) | 1.3 (-2, 4.7) | 0.988 |
|  | Lag3 | 0.7 (-1.7, 3.1) | -0.1 (-2.2, 2.1) | 0.616 | -1.5 (-4.9, 1.9) | -2.9 (-6.3, 0.7) | 0.522 |
|  | Lag4 | 0.8 (-1.8, 3.4) | -0.1 (-2.2, 2.1) | 0.578 | -0.9 (-4, 2.4) | -3.2 (-6.2, 0) | 0.246 |
|  | 5dMA | 0 (-3.6, 3.8) | 0.8 (-2.6, 4.3) | 0.723 | 1.7 (-4.4, 8.2) | -2.5 (-7.6, 2.8) | 0.156 |
| sVCAM-1 | Lag0 | 0.7 (-2.1, 3.6) | 1.4 (-1.4, 4.2) | 0.717 | 1.9 (-1.9, 5.8) | -2.5 (-5.8, 1) | 0.053 |
|  | Lag1 | 0.9 (-1.7, 3.6) | 1.4 (-1.1, 4) | 0.766 | 1.6 (-2.3, 5.6) | -0.9 (-4.2, 2.5) | 0.274 |
|  | Lag2 | 0.4 (-2.1, 3.1) | -0.6 (-3, 1.8) | 0.518 | 0.9 (-3, 4.9) | -0.9 (-4.3, 2.7) | 0.446 |
|  | Lag3 | 2.6 (0.1, 5.3) * | -0.3 (-2.5, 2) | 0.079 | -2.3 (-5.8, 1.4) | -3.8 (-7.4, -0.1) * | 0.496 |
|  | Lag4 | 2.1 (-0.7, 4.9) | -0.3 (-2.6, 2) | 0.148 | -1.4 (-4.8, 2.1) | -2.4 (-5.7, 1.1) | 0.665 |
|  | 5dMA | 3.1 (-0.9, 7.2) | 0.5 (-3.1, 4.1) | 0.291 | -0.8 (-7.1, 5.9) | -4.5 (-9.6, 0.9) | 0.238 |
| SDNN | Lag0 | -2.5 (-7.3, 2.7) | -3.6 (-8.3, 1.4) | 0.738 | 1.7 (-5.1, 9) | 2.8 (-3.6, 9.5) | 0.798 |
|  | Lag1 | -1.2 (-6, 3.7) | -2.2 (-6.7, 2.5) | 0.764 | 2.3 (-4.7, 9.9) | 0.4 (-5.7, 6.8) | 0.641 |
|  | Lag2 | 4.3 (-0.6, 9.5) | 0.7 (-3.6, 5.1) | 0.246 | 0.3 (-6.6, 7.8) | -2.8 (-8.8, 3.6) | 0.454 |
|  | Lag3 | 3.2 (-1.5, 8.1) | 0 (-4.1, 4.3) | 0.304 | 1.4 (-5.1, 8.3) | -2.2 (-8.6, 4.8) | 0.404 |
|  | Lag4 | 0.8 (-4.1, 5.9) | -1.3 (-5.3, 2.9) | 0.488 | 3 (-3.4, 9.8) | 4.6 (-1.7, 11.3) | 0.702 |
|  | 5dMA | 2.6 (-4.6, 10.3) | -1.9 (-8.2, 4.7) | 0.312 | 6.5 (-5.7, 20.2) | 2.7 (-7, 13.6) | 0.551 |
| RMSSD | Lag0 | -5 (-12.1, 2.6) | -4.4 (-11.4, 3.2) | 0.893 | 0.4 (-9.6, 11.6) | 2.4 (-7, 12.7) | 0.754 |
|  | Lag1 | -2.2 (-9.1, 5.3) | -1.8 (-8.5, 5.3) | 0.932 | 5.6 (-5.2, 17.5) | 2.7 (-6.4, 12.7) | 0.662 |
|  | Lag2 | 4.1 (-3.1, 12) | 0.8 (-5.5, 7.6) | 0.48 | 0 (-10.3, 11.4) | -1.4 (-10.4, 8.5) | 0.829 |
|  | Lag3 | 2.5 (-4.5, 10) | -0.1 (-6.3, 6.4) | 0.568 | 3.5 (-6.4, 14.4) | -0.1 (-9.9, 10.8) | 0.579 |
|  | Lag4 | -1.1 (-8.2, 6.6) | -1.7 (-7.7, 4.7) | 0.89 | 4.1 (-5.4, 14.7) | 8.4 (-1.2, 19) | 0.500 |
|  | 5dMA | 0 (-10.3, 11.5) | -1.9 (-11.1, 8.3) | 0.778 | 10.8 (-7.6, 32.9) | 7.8 (-7.2, 25.3) | 0.758 |
| HFn | Lag0 | -3.2 (-10.4, 4) | -6.3 (-13.5, 0.8) | 0.509 | 2.5 (-7.4, 12.3) | 2.3 (-6.7, 11.2) | 0.972 |
|  | Lag1 | -1.7 (-8.5, 5.2) | -3.6 (-10.1, 3) | 0.669 | 3.8 (-6.2, 13.7) | 1 (-7.7, 9.7) | 0.634 |
|  | Lag2 | 0.4 (-6.4, 7.2) | -2.5 (-8.6, 3.6) | 0.498 | -3.3 (-13.5, 6.9) | 0.7 (-8.3, 9.6) | 0.510 |
|  | Lag3 | 0 (-6.6, 6.5) | -2.6 (-8.4, 3.3) | 0.55 | 0.2 (-9.1, 9.4) | 6.8 (-2.8, 16.3) | 0.262 |
|  | Lag4 | -0.8 (-7.8, 6.2) | 0.3 (-5.6, 6.2) | 0.798 | -0.1 (-9, 8.9) | 5.4 (-3.3, 14) | 0.325 |
|  | 5dMA | -2.6 (-12.8, 7.5) | -5.9 (-15.1, 3.3) | 0.593 | 3.3 (-13.6, 20.1) | 6.8 (-7.2, 20.7) | 0.669 |
| LFn | Lag0 | 3.6 (-2.3, 9.5) | 5.9 (0.1, 11.8) * | 0.543 | -2.8 (-10.7, 5.2) | -3.6 (-10.9, 3.7) | 0.857 |
|  | Lag1 | 1 (-4.5, 6.5) | 4.1 (-1.1, 9.4) | 0.375 | -4.5 (-12.5, 3.5) | -1.6 (-8.6, 5.3) | 0.538 |
|  | Lag2 | 1.8 (-3.6, 7.3) | 3.2 (-1.7, 8.1) | 0.694 | -1.1 (-9.3, 7) | -1.5 (-8.7, 5.6) | 0.931 |
|  | Lag3 | 1 (-4.3, 6.4) | -0.5 (-5.3, 4.3) | 0.655 | 0.7 (-6.9, 8.2) | -4.3 (-12, 3.4) | 0.298 |
|  | Lag4 | 1.9 (-3.8, 7.5) | -1.8 (-6.6, 2.9) | 0.276 | -0.2 (-7.5, 7) | -5.2 (-12.1, 1.8) | 0.265 |
|  | 5dMA | 3.9 (-4.3, 12.1) | 3.9 (-3.6, 11.3) | 0.996 | -5.7 (-19.3, 8) | -7.1 (-18.4, 4.2) | 0.825 |
|  |  |  |  |  |  |  |  |
|  |  |  |  |  |  |  |  |
|  |  |  |  |  |  |  |  |
|  |  |  |  |  |  |  |  |
|  |  |  |  |  |  |  |  |
| Supplemental Table 7 continued | | | | | | | |
| LF/HF | Lag0 | 8 (-6.1, 24.2) | 16.4 (1.3, 33.6) * | 0.417 | -5.1 (-21.5, 14.7) | -6.7 (-21.5, 11) | 0.878 |
|  | Lag1 | 3.4 (-9.4, 18) | 9.6 (-3.4, 24.4) | 0.491 | -10.4 (-26.1, 8.6) | -2.4 (-17.5, 15.4) | 0.445 |
|  | Lag2 | 2.5 (-10.1, 16.9) | 6.4 (-5.5, 19.7) | 0.658 | 2.4 (-15.8, 24.7) | -2.5 (-18, 15.9) | 0.664 |
|  | Lag3 | 1.3 (-10.8, 15) | 2.8 (-8.3, 15.2) | 0.857 | 0.6 (-15.9, 20.4) | -11.7 (-26.5, 6.1) | 0.252 |
|  | Lag4 | 4.3 (-8.9, 19.3) | -1.8 (-12.3, 9.9) | 0.458 | -1.2 (-16.8, 17.4) | -11.5 (-25, 4.5) | 0.302 |
|  | 5dMA | 8.8 (-10.5, 32.3) | 13 (-5.4, 34.9) | 0.755 | -10.4 (-35.3, 24) | -14.4 (-34.6, 12.1) | 0.773 |
| VLF | Lag0 | -17.8 (-33.4, 1.4) | -12.4 (-29.1, 8.3) | 0.648 | -16 (-36.9, 11.6) | 6.9 (-17.5, 38.5) | 0.139 |
|  | Lag1 | -7.7 (-24.3, 12.6) | -13 (-28.1, 5.2) | 0.639 | 13 (-15.4, 50.8) | 12.5 (-12.6, 44.7) | 0.980 |
|  | Lag2 | -4.7 (-21.7, 16.1) | -14.8 (-28.6, 1.8) | 0.37 | 25.5 (-6.6, 68.8) | 11.1 (-14.5, 44.3) | 0.470 |
|  | Lag3 | 11.4 (-7.9, 34.7) | 8.8 (-8.3, 29.1) | 0.85 | 3 (-21.5, 35.3) | -19.9 (-39.4, 6) | 0.134 |
|  | Lag4 | 0 (-18.5, 22.8) | 0.9 (-15.3, 20.2) | 0.941 | -5.2 (-26.9, 23) | -6.7 (-27.7, 20.2) | 0.916 |
|  | 5dMA | -7 (-30.7, 24.8) | -12.4 (-33.2, 15) | 0.743 | 1.2 (-38.6, 66.8) | -3.9 (-36.8, 46.2) | 0.824 |
| P Complexity | Lag0 | 1.8 (-5.4, 9.5) | 2.5 (-4.8, 10.3) | 0.889 | 0.3 (-9.3, 10.9) | 0.3 (-8.4, 9.9) | 0.998 |
|  | Lag1 | 0.7 (-5.9, 7.7) | 4.9 (-1.7, 11.9) | 0.345 | -0.3 (-9.7, 10.1) | -1.5 (-9.6, 7.3) | 0.827 |
|  | Lag2 | 4.6 (-2, 11.7) | 5.9 (-0.2, 12.3) | 0.774 | -8.7 (-17.2, 0.8) | -6.4 (-14.2, 2.1) | 0.671 |
|  | Lag3 | 3.6 (-3.1, 10.6) | 2.9 (-3, 9.1) | 0.877 | -5.9 (-14.3, 3.3) | -2.8 (-11.7, 6.9) | 0.591 |
|  | Lag4 | -1.5 (-8.1, 5.5) | 7.7 (1.6, 14.1) * | 0.032 | -5.1 (-13.1, 3.8) | 4.2 (-4.5, 13.7) | 0.092 |
|  | 5dMA | 4.5 (-5.5, 15.5) | 13.2 (3.3, 24.1) * | 0.191 | -9.9 (-23.8, 6.6) | -3.9 (-16.3, 10.4) | 0.439 |
| QRS | Lag0 | 2.6 (-3.6, 9.1) | 3.8 (-2.3, 10.2) | 0.776 | 0.9 (-7.2, 9.8) | 0.1 (-7.3, 8) | 0.861 |
|  | Lag1 | 0.7 (-5, 6.7) | 1.8 (-3.7, 7.5) | 0.775 | -5.9 (-13.6, 2.4) | -4 (-10.8, 3.3) | 0.687 |
|  | Lag2 | 0.2 (-5.3, 5.9) | 3.2 (-1.9, 8.5) | 0.403 | -8.9 (-16.2, -0.9) * | -3.3 (-10.1, 4.2) | 0.227 |
|  | Lag3 | 0.8 (-4.4, 6.4) | 2.3 (-2.5, 7.3) | 0.673 | -10.2 (-16.7, -3.1) * | -4.8 (-12, 3) | 0.234 |
|  | Lag4 | 1.2 (-4.6, 7.4) | -0.2 (-5, 4.9) | 0.686 | 1.3 (-6.2, 9.4) | 2.2 (-5.1, 10.1) | 0.855 |
|  | 5dMA | 1.5 (-6.8, 10.7) | 4.6 (-3.2, 13) | 0.576 | -12.3 (-24.1, 1.2) | -6.2 (-16.7, 5.7) | 0.340 |
| QTc | Lag0 | 0.4 (-0.2, 1.1) | 0.3 (-0.3, 0.9) | 0.784 | 0.5 (-0.4, 1.3) | 0.1 (-0.6, 0.9) | 0.487 |
|  | Lag1 | 0.4 (-0.2, 1) | 0.2 (-0.4, 0.8) | 0.644 | 0.5 (-0.4, 1.4) | -0.3 (-1, 0.5) | 0.122 |
|  | Lag2 | 0.2 (-0.4, 0.8) | 0.2 (-0.4, 0.7) | 0.961 | 0.1 (-0.8, 1) | -0.2 (-0.9, 0.6) | 0.640 |
|  | Lag3 | 0.4 (-0.2, 0.9) | 0.3 (-0.2, 0.8) | 0.888 | 0.2 (-0.6, 1.1) | -0.4 (-1.2, 0.5) | 0.233 |
|  | Lag4 | 0.3 (-0.3, 0.9) | -0.2 (-0.7, 0.3) | 0.126 | 0.4 (-0.4, 1.2) | -0.2 (-0.9, 0.6) | 0.255 |
|  | 5dMA | 0.7 (-0.2, 1.6) | 0.4 (-0.4, 1.2) | 0.539 | 1 (-0.5, 2.5) | -0.1 (-1.3, 1.1) | 0.126 |
| T Complexity | Lag0 | 3.1 (-3.5, 10.2) | -0.1 (-6.4, 6.7) | 0.468 | -2.8 (-11.2, 6.4) | 4 (-4.2, 12.9) | 0.205 |
|  | Lag1 | 5.8 (-0.7, 12.7) | 3.1 (-2.9, 9.5) | 0.528 | -7.4 (-15.5, 1.5) | 0.5 (-7.2, 8.8) | 0.130 |
|  | Lag2 | 4.1 (-2.2, 10.8) | -2.3 (-7.6, 3.3) | 0.104 | -3.5 (-12.1, 6) | 0.2 (-7.7, 8.9) | 0.497 |
|  | Lag3 | 4 (-2, 10.4) | -1 (-6.1, 4.5) | 0.205 | -3 (-10.9, 5.6) | -0.6 (-9, 8.6) | 0.655 |
|  | Lag4 | -2.2 (-8.3, 4.3) | -2.7 (-7.8, 2.7) | 0.891 | -0.3 (-8.3, 8.3) | 6.4 (-1.8, 15.3) | 0.206 |
|  | 5dMA | 5.7 (-3.7, 16.1) | -1.1 (-9.1, 7.6) | 0.242 | -7.8 (-21.2, 7.8) | 3.6 (-9, 17.9) | 0.132 |

Note: the effect estimates of biomarkers per IQR increase in one pollutant were adjusted by the other one. * *p* < 0.05 for significant association within a group.

**Supplemental Table 8**. Percent change (95% CI) in biomarkers per IQR increase in PM_2.5_ or O_3_ in the low and high omega-3 groups after controlling for NO_2_ of the same lag.

| Outcome | Lag (Day) | Effects of PM_2.5_ adjusted for NO_2_ | | | |  | Effects of O_3_ adjusted for NO_2_ | | |
| --- | --- | --- | --- | --- | --- | --- | --- | --- | --- |
|  |  | Low | High | *p*_interaction_ | |  | Low | High | *p*_interaction_ |
| Total cholesterol | Lag0 | 0 (-2.3, 2.3) | 0.8 (-1.3, 3) | 0.578 | |  | 4 (0.9, 7.1) * | -0.5 (-3.2, 2.3) | 0.016 |
|  | Lag1 | -3.4 (-5.5, -1.2) * | -1.1 (-3.2, 1) | 0.091 | |  | 0.6 (-2.3, 3.6) | -2.9 (-5.4, -0.4) * | 0.046 |
|  | Lag2 | -1.7 (-3.6, 0.2) | -1.1 (-2.8, 0.7) | 0.627 | |  | -2.8 (-5.5, 0) * | -1.4 (-4, 1.2) | 0.442 |
|  | Lag3 | -1.4 (-3.3, 0.6) | -0.1 (-1.9, 1.8) | 0.313 | |  | -2.7 (-5.4, 0.1) | -0.3 (-3.1, 2.7) | 0.180 |
|  | Lag4 | -1.6 (-3.6, 0.4) | -0.8 (-2.6, 0.9) | 0.554 | |  | -2.9 (-5.2, -0.4) * | 0.7 (-2, 3.3) | 0.046 |
|  | 5dMA | -3.7 (-6.7, -0.6) * | -1.5 (-4.2, 1.3) | 0.234 | |  | -3.7 (-8.4, 1.2) | -3.4 (-7.3, 0.7) | 0.889 |
| HDL | Lag0 | -2 (-4.5, 0.4) | 0 (-2.3, 2.4) | 0.182 | |  | 2 (-1.3, 5.4) | 0 (-3.1, 3.1) | 0.314 |
|  | Lag1 | -2.7 (-5.1, -0.3) * | -1.8 (-4, 0.5) | 0.511 | |  | -2.4 (-5.5, 0.8) | -1.3 (-4, 1.5) | 0.575 |
|  | Lag2 | -1 (-3.1, 1.2) | -0.8 (-2.7, 1.2) | 0.874 | |  | -2.8 (-5.8, 0.3) | -0.4 (-3.2, 2.5) | 0.218 |
|  | Lag3 | -1 (-3.1, 1.3) | -0.2 (-2.2, 1.8) | 0.613 | |  | -2.8 (-5.8, 0.2) | 1.7 (-1.5, 5) | 0.024 |
|  | Lag4 | 0.1 (-2.1, 2.4) | -2.2 (-4.1, -0.2) * | 0.113 | |  | -2.8 (-5.5, -0.1) * | 1.1 (-1.8, 4.1) | 0.047 |
|  | 5dMA | -2.8 (-6, 0.6) | -2.3 (-5.2, 0.7) | 0.813 | |  | -5.6 (-10.6, -0.3) * | -1.1 (-5.5, 3.5) | 0.100 |
| LDL | Lag0 | 1.3 (-2, 4.7) | 0.3 (-2.7, 3.4) | 0.621 | |  | 3.7 (-0.5, 8.2) | -2.5 (-6.3, 1.3) | 0.015 |
|  | Lag1 | -3.9 (-6.9, -0.7) * | -2.5 (-5.4, 0.5) | 0.460 | |  | 0.4 (-3.8, 4.7) | -5.3 (-8.7, -1.7) * | 0.023 |
|  | Lag2 | -1.6 (-4.4, 1.2) | -1.2 (-3.7, 1.4) | 0.831 | |  | -4.2 (-8.1, -0.2) * | -1.4 (-5.1, 2.4) | 0.259 |
|  | Lag3 | -1.5 (-4.3, 1.4) | 1.2 (-1.4, 3.9) | 0.158 | |  | -3.1 (-7, 0.8) | 0.1 (-4, 4.3) | 0.215 |
|  | Lag4 | -1 (-3.9, 2) | 0 (-2.5, 2.6) | 0.604 | |  | -3.7 (-7.1, -0.2) * | 1.9 (-1.9, 5.8) | 0.027 |
|  | 5dMA | -3.4 (-7.7, 1.1) | -1.5 (-5.4, 2.6) | 0.477 | |  | -5.9 (-12.4, 1.1) | -5.1 (-10.7, 0.8) | 0.824 |
| Triglycerides | Lag0 | -2.8 (-10, 5) | 3.4 (-3.7, 11) | 0.204 | |  | 2 (-7.7, 12.7) | 4.7 (-4.6, 14.9) | 0.668 |
|  | Lag1 | -1.7 (-8.9, 6.2) | 8.5 (0.8, 16.7) * | 0.036 | |  | 3.7 (-6.4, 14.9) | 1.2 (-7.5, 10.7) | 0.692 |
|  | Lag2 | -2.2 (-8.7, 4.9) | -2.8 (-8.7, 3.5) | 0.886 | |  | 2.7 (-7.1, 13.6) | -4.2 (-12.6, 4.9) | 0.256 |
|  | Lag3 | -0.7 (-7.3, 6.4) | -4.3 (-10, 1.8) | 0.403 | |  | -0.4 (-9.5, 9.6) | -6.4 (-15.1, 3.2) | 0.315 |
|  | Lag4 | -4.4 (-10.9, 2.5) | 0.6 (-5.3, 6.9) | 0.252 | |  | 2.3 (-6.1, 11.5) | -5.7 (-13.7, 3) | 0.172 |
|  | 5dMA | -4.2 (-14, 6.7) | 0.8 (-8.5, 11.1) | 0.429 | |  | 5.3 (-11.2, 25) | -3.2 (-16.1, 11.7) | 0.327 |
| tPA | Lag0 | -2.1 (-7.8, 3.9) | 0.5 (-4.8, 6.1) | 0.480 | |  | -0.6 (-7.9, 7.2) | 2 (-5, 9.6) | 0.573 |
|  | Lag1 | -3 (-8.5, 2.9) | -0.3 (-5.6, 5.3) | 0.431 | |  | 0.7 (-6.8, 8.8) | -0.9 (-7.3, 6) | 0.734 |
|  | Lag2 | 2.9 (-2.2, 8.2) | -1.3 (-5.7, 3.3) | 0.190 | |  | -1.9 (-9, 5.8) | 2 (-4.6, 9.2) | 0.394 |
|  | Lag3 | 6.1 (0.8, 11.7) * | 0.9 (-3.6, 5.7) | 0.133 | |  | 2.3 (-4.8, 9.9) | 6.4 (-1.1, 14.5) | 0.401 |
|  | Lag4 | -3.1 (-8.1, 2.2) | 2.1 (-2.5, 6.8) | 0.123 | |  | -0.8 (-7.1, 5.9) | 3 (-3.6, 10) | 0.409 |
|  | 5dMA | 0.4 (-7.5, 8.9) | 1.3 (-5.7, 8.8) | 0.852 | |  | 1.7 (-10.6, 15.6) | 6.9 (-3.9, 19) | 0.445 |
| vWF | Lag0 | 6.1 (0.6, 11.9) * | -0.2 (-4.9, 4.6) | 0.060 | |  | 12.9 (5.7, 20.6) * | -5.4 (-11.1, 0.6) | 0.000 |
|  | Lag1 | -2.4 (-7.4, 2.8) | -1.9 (-6.5, 3) | 0.863 | |  | 5.1 (-1.8, 12.5) | -6.6 (-11.8, -0.9) * | 0.004 |
|  | Lag2 | -5.6 (-9.8, -1.1) * | -3.4 (-7.3, 0.6) | 0.433 | |  | -2.3 (-8.8, 4.6) | -5.5 (-11.1, 0.4) | 0.432 |
|  | Lag3 | -6.6 (-10.9, -2.1)* | -1.4 (-5.4, 2.8) | 0.072 | |  | -1 (-7.4, 5.8) | -6.8 (-12.9, -0.4) * | 0.158 |
|  | Lag4 | -4.9 (-9.5, -0.2) * | -0.4 (-4.5, 3.8) | 0.133 | |  | -1 (-6.8, 5.1) | 0.3 (-5.6, 6.6) | 0.752 |
|  | 5dMA | -7.9 (-14.3, -1) * | -4.2 (-10.2, 2.1) | 0.369 | |  | 5 (-6.5, 17.8) | -9.6 (-17.8, -0.6) * | 0.012 |
| D-dimer | Lag0 | 3.1 (-8.7, 16.3) | -2.9 (-13.1, 8.5) | 0.428 | |  | 8.3 (-7.4, 26.7) | 7.2 (-7.3, 24) | 0.915 |
|  | Lag1 | 10.4 (-2.9, 25.6) | -2.7 (-13.8, 9.9) | 0.106 | |  | 14.7 (-3.2, 36) | 2.8 (-11.4, 19.3) | 0.275 |
|  | Lag2 | 3.2 (-8.2, 16.1) | -4.2 (-13.7, 6.4) | 0.310 | |  | 9.4 (-7.7, 29.6) | 0.8 (-13.4, 17.3) | 0.421 |
|  | Lag3 | -6.7 (-17, 4.9) | -5.7 (-15.1, 4.7) | 0.888 | |  | 9.6 (-7.0, 29.0) | 2.6 (-12.7, 20.6) | 0.526 |
|  | Lag4 | -11.9 (-21.7, -0.8) * | -8.7 (-17.5, 1.2) | 0.632 | |  | -5.2 (-18, 9.6) | 0.3 (-13.2, 15.9) | 0.562 |
|  | 5dMA | -3.6 (-19.6, 15.5) | -12 (-25.3, 3.6) | 0.403 | |  | 26 (-5.2, 67.4) | 12.9 (-10.9, 43.1) | 0.432 |
|  |  |  |  |  | |  |  |  |  |
|  |  |  |  |  | |  |  |  |  |
| Supplemental Table 8 continued | | | | | | | | | |
| SAA | Lag0 | 9.4 (-6.8, 28.5) | 3 (-11.1, 19.3) | 0.544 | |  | 7.9 (-12.4, 32.9) | 1.2 (-16.6, 23) | 0.615 |
|  | Lag1 | 8.2 (-8.3, 27.5) | -4.7 (-18.4, 11.2) | 0.203 | |  | 23.4 (-0.5, 53.1) | 2.2 (-15.3, 23.3) | 0.144 |
|  | Lag2 | -5.7 (-18.3, 8.8) | -3.8 (-15.4, 9.4) | 0.819 | |  | 9.1 (-11.7, 34.6) | -5 (-21.3, 14.8) | 0.284 |
|  | Lag3 | 2.1 (-11.9, 18.2) | -8 (-19.4, 5.0) | 0.273 | |  | 7.1 (-12.8, 31.6) | 3.7 (-15.7, 27.6) | 0.806 |
|  | Lag4 | 6.6 (-8.3, 24) | 0.2 (-12, 14.1) | 0.515 | |  | -1.5 (-18, 18.5) | 6.1 (-11.9, 27.7) | 0.560 |
|  | 5dMA | 6 (-15.7, 33.2) | -8.3 (-25.2, 12.5) | 0.295 | |  | 28.8 (-10.3, 84.9) | 8.9 (-19.4, 47.1) | 0.359 |
| sICAM1 | Lag0 | 1.4 (-1.1, 4.0) | 1.3 (-1, 3.6) | 0.920 | |  | 4.5 (1.3, 7.8) * | 1.3 (-1.5, 4.3) | 0.101 |
|  | Lag1 | 1.0 (-1.5, 3.5) | 2.4 (0.1, 4.8) * | 0.340 | |  | 1.4 (-1.9, 4.7) | 0.1 (-2.7, 3.0) | 0.514 |
|  | Lag2 | -1.1 (-3.3, 1.1) | 0.4 (-1.6, 2.4) | 0.258 | |  | -0.2 (-3.4, 3.1) | 1.2 (-1.7, 4.2) | 0.478 |
|  | Lag3 | -0.9 (-3.1, 1.3) | -0.6 (-2.6, 1.4) | 0.843 | |  | -2.4 (-5.3, 0.7) | -2.7 (-5.7, 0.4) | 0.872 |
|  | Lag4 | -0.5 (-2.7, 1.8) | -0.7 (-2.6, 1.3) | 0.915 | |  | -1 (-3.7, 1.8) | -2.4 (-5.1, 0.3) | 0.436 |
|  | 5dMA | -0.5 (-3.9, 3.0) | 0.7 (-2.4, 3.9) | 0.580 | |  | -0.1 (-5.6, 5.6) | -2.3 (-6.8, 2.4) | 0.426 |
| sVCAM1 | Lag0 | 1.9 (-0.5, 4.3) | 1.5 (-0.6, 3.7) | 0.812 | |  | 1.6 (-1.5, 4.8) | -1.6 (-4.4, 1.3) | 0.089 |
|  | Lag1 | 1.0 (-1.4, 3.4) | 1.4 (-0.8, 3.6) | 0.763 | |  | 0.7 (-2.4, 3.9) | -0.5 (-3.2, 2.2) | 0.502 |
|  | Lag2 | 0.7 (-1.4, 2.8) | -0.2 (-2, 1.7) | 0.514 | |  | -0.6 (-3.6, 2.5) | -0.1 (-2.8, 2.7) | 0.787 |
|  | Lag3 | 0.8 (-1.3, 2.9) | -0.6 (-2.4, 1.3) | 0.323 | |  | -2.8 (-5.6, 0.1) | -2.5 (-5.3, 0.5) | 0.857 |
|  | Lag4 | 0.4 (-1.7, 2.6) | -1 (-2.8, 0.9) | 0.302 | |  | -1.2 (-3.8, 1.4) | -1.6 (-4.2, 1.1) | 0.859 |
|  | 5dMA | 1.8 (-1.5, 5.2) | 0.3 (-2.6, 3.2) | 0.434 | |  | -2.5 (-7.4, 2.7) | -3.1 (-7.2, 1.1) | 0.799 |
| SDNN | Lag0 | -2.1 (-7.3, 3.3) | -1.7 (-6.5, 3.2) | 0.901 | |  | 0.6 (-6.2, 8.0) | 1.6 (-4.8, 8.4) | 0.821 |
|  | Lag1 | -0.9 (-6.2, 4.6) | -1.6 (-6.4, 3.4) | 0.835 | |  | 2.1 (-5.0, 9.7) | -0.1 (-6, 6.2) | 0.609 |
|  | Lag2 | 4.2 (-0.7, 9.3) | -0.2 (-4.3, 4.2) | 0.150 | |  | 1.8 (-5.1, 9.2) | -1.5 (-7.4, 4.8) | 0.444 |
|  | Lag3 | 3.3 (-1.5, 8.3) | 0.1 (-4.1, 4.5) | 0.309 | |  | 2.2 (-4.3, 9.2) | -1.1 (-7.5, 5.6) | 0.433 |
|  | Lag4 | 1.7 (-3.1, 6.7) | 0.2 (-3.8, 4.4) | 0.633 | |  | 2.4 (-3.4, 8.6) | 5.3 (-0.6, 11.7) | 0.487 |
|  | 5dMA | 3.3 (-4.2, 11.5) | -1.2 (-7.6, 5.5) | 0.313 | |  | 6.4 (-5.6, 20.0) | 2.8 (-6.7, 13.2) | 0.565 |
| RMSSD | Lag0 | -4.0 (-11.4, 4.1) | -2.1 (-9.1, 5.4) | 0.704 | |  | -1.7 (-11.5, 9.3) | -0.3 (-9.6, 9.8) | 0.835 |
|  | Lag1 | -2.5 (-10.1, 5.7) | -1.3 (-8.4, 6.3) | 0.795 | |  | 4.3 (-6.3, 16.1) | 2.0 (-6.9, 11.7) | 0.725 |
|  | Lag2 | 3.2 (-4.0, 10.9) | -0.2 (-6.3, 6.4) | 0.453 | |  | 1.0 (-9.2, 12.2) | 0.2 (-8.7, 10.0) | 0.910 |
|  | Lag3 | 2.3 (-4.8, 9.9) | -0.4 (-6.6, 6.3) | 0.565 | |  | 4.1 (-5.8, 15.0) | 0.7 (-8.9, 11.4) | 0.614 |
|  | Lag4 | -0.1 (-6.9, 7.1) | -0.4 (-6.2, 5.8) | 0.959 | |  | 2.4 (-6.0, 11.6) | 6.4 (-2.3, 15.9) | 0.518 |
|  | 5dMA | -0.2 (-10.9, 11.8) | -1.5 (-10.8, 8.8) | 0.848 | |  | 9.1 (-8.9, 30.6) | 6.8 (-7.6, 23.5) | 0.816 |
| HFn | Lag0 | -3.5 (-11.0, 3.9) | -4.9 (-11.7, 2.0) | 0.776 | |  | 0.3 (-9.7, 10.2) | 0.3 (-8.9, 9.5) | 0.997 |
|  | Lag1 | 0.8 (-6.6, 8.3) | -1.5 (-8.5, 5.4) | 0.594 | |  | 3.3 (-6.7, 13.2) | 0.5 (-7.9, 9.0) | 0.642 |
|  | Lag2 | 0.6 (-6.2, 7.3) | -2.2 (-8.1, 3.7) | 0.509 | |  | -2.8 (-12.6, 6.9) | 0.0 (-8.6, 8.6) | 0.631 |
|  | Lag3 | 2.2 (-4.4, 8.8) | -0.8 (-6.7, 5.2) | 0.478 | |  | 1.2 (-7.9, 10.3) | 6.7 (-2.4, 15.9) | 0.341 |
|  | Lag4 | 1.1 (-5.5, 7.8) | 2.5 (-3.2, 8.3) | 0.745 | |  | -0.2 (-8.4, 8.0) | 6.0 (-2.2, 14.1) | 0.270 |
|  | 5dMA | 1.5 (-8.9, 12) | -3.1 (-12.3, 6.0) | 0.453 | |  | 4.1 (-12.4, 20.7) | 5.1 (-8.3, 18.4) | 0.910 |
| LFn | Lag0 | 2.7 (-3.3, 8.8) | 5.2 (-0.4, 10.7) | 0.521 | |  | -1.1 (-9.1, 7.0) | -1.4 (-8.8, 6.0) | 0.954 |
|  | Lag1 | -0.7 (-6.8, 5.3) | 2.6 (-3.0, 8.2) | 0.358 | |  | -3.9 (-11.8, 4.1) | -1.3 (-8.1, 5.4) | 0.592 |
|  | Lag2 | 1.3 (-4.2, 6.8) | 1.4 (-3.5, 6.2) | 0.975 | |  | -0.4 (-8.3, 7.5) | -0.3 (-7.2, 6.7) | 0.974 |
|  | Lag3 | -0.5 (-5.9, 4.9) | -1.5 (-6.3, 3.4) | 0.784 | |  | 0.0 (-7.5, 7.5) | -4.5 (-12, 2.9) | 0.343 |
|  | Lag4 | 0.8 (-4.6, 6.2) | -3.5 (-8.1, 1.2) | 0.209 | |  | -0.2 (-6.7, 6.4) | -6.6 (-13.1, -0.1) * | 0.150 |
|  | 5dMA | 0.9 (-7.6, 9.4) | 1.7 (-5.8, 9.2) | 0.873 | |  | -5.8 (-19.3, 7.6) | -5.5 (-16.4, 5.4) | 0.965 |
| LF/HF | Lag0 | 7.9 (-6.6, 24.6) | 13.1 (-0.9, 29.1) | 0.597 | |  | -0.7 (-18.1, 20.4) | -1.8 (-17.7, 17.3) | 0.924 |
|  | Lag1 | -1.4 (-14.7, 13.9) | 5.0 (-8.2, 20.0) | 0.468 | |  | -9.1 (-25.0, 10.1) | -1.6 (-16.4, 15.9) | 0.484 |
|  | Lag2 | 1.7 (-10.8, 15.9) | 4.4 (-7.0, 17.1) | 0.747 | |  | 3.1 (-14.7, 24.6) | -0.5 (-15.7, 17.6) | 0.760 |
|  | Lag3 | -3.2 (-14.8, 10.0) | -0.6 (-11.4, 11.6) | 0.744 | |  | -1.2 (-17.2, 17.9) | -11.8 (-26.1, 5.3) | 0.316 |
|  | Lag4 | 0.5 (-11.6, 14.3) | -6.2 (-16.1, 4.7) | 0.389 | |  | -0.3 (-14.8, 16.7) | -12.9 (-25.5, 1.8) | 0.208 |
|  | 5dMA | 0.3 (-18.0, 22.7) | 6.7 (-10.6, 27.4) | 0.604 | |  | -11.0 (-35.4, 22.5) | -10.7 (-31.1, 15.6) | 0.984 |
| Supplemental Table 8 continued | | | | | | | | | |
| VLF | Lag0 | -15.3 (-31.7, 5.1) | -10.8 (-27.1, 9.1) | 0.710 | |  | -17.4 (-38.1, 10.2) | -2.0 (-24.5, 27.4) | 0.306 |
|  | Lag1 | -1.8 (-20.9, 21.9) | -8 (-24.9, 12.7) | 0.621 | |  | 11.1 (-16.6, 48.0) | 9.5 (-14.4, 39.9) | 0.928 |
|  | Lag2 | 1.5 (-16.8, 23.8) | -9.6 (-24.0, 7.5) | 0.346 | |  | 21.9 (-8.4, 62.1) | 6.3 (-17.3, 36.7) | 0.420 |
|  | Lag3 | 7.7 (-11.4, 30.9) | 4.9 (-11.9, 25.0) | 0.834 | |  | 5.9 (-19.0, 38.5) | -16.5 (-36.3, 9.6) | 0.155 |
|  | Lag4 | -8.1 (-24.4, 11.8) | -3.1 (-18.1, 14.7) | 0.669 | |  | -5.0 (-25.1, 20.4) | -3.3 (-23.9, 22.9) | 0.909 |
|  | 5dMA | -8.5 (-32.7, 24.4) | -13.2 (-34.1, 14.4) | | 0.774 |  | -1.5 (-40.1, 62.0) | -7.7 (-38.7, 39.2) | 0.783 |
| P Complexity | Lag0 | 1.2 (-6.1, 9.1) | 1.7 (-5.2, 9.0) | 0.919 | |  | -0.2 (-9.8, 10.5) | -0.1 (-8.9, 9.6) | 0.987 |
|  | Lag1 | -0.2 (-7.4, 7.6) | 4.7 (-2.3, 12.3) | 0.288 | |  | -0.2 (-9.6, 10.2) | 0.1 (-8.0, 8.9) | 0.961 |
|  | Lag2 | 3.7 (-3.0, 10.8) | 6.4 (0.4, 12.8) * | 0.529 | |  | -5.5 (-14.1, 4.0) | -3.7 (-11.6, 4.8) | 0.754 |
|  | Lag3 | 1.8 (-4.7, 8.8) | 1.7 (-4.1, 7.9) | 0.981 | |  | -5.6 (-14.0, 3.6) | -1.7 (-10.4, 7.9) | 0.496 |
|  | Lag4 | -1.7 (-8.1, 5.1) | 8.1 (2.1, 14.5) * | 0.024 | |  | -3.1 (-10.8, 5.3) | 7.6 (-1.1, 17.0) | 0.069 |
|  | 5dMA | 3.3 (-7.0, 14.7) | 12.2 (2.4, 23.1) * | 0.176 | |  | -7.4 (-21.6, 9.4) | 0.4 (-12.4, 14.9) | 0.343 |
| QRS | Lag0 | 1.3 (-4.9, 7.9) | 3.4 (-2.4, 9.5) | 0.595 | |  | 1.3 (-6.9, 10.2) | -1.4 (-8.8, 6.6) | 0.601 |
|  | Lag1 | -3.4 (-9.3, 3.0) | -1.4 (-7.0, 4.5) | 0.595 | |  | -6.3 (-13.8, 1.9) | -4.8 (-11.3, 2.2) | 0.742 |
|  | Lag2 | -1.9 (-7.2, 3.8) | 0.7 (-4.1, 5.8) | 0.449 | |  | -6.8 (-14.2, 1.2) | -2.3 (-9.2, 5.1) | 0.353 |
|  | Lag3 | -1.1 (-6.4, 4.5) | 0.7 (-4.2, 5.9) | 0.607 | |  | -9.1 (-15.9, -1.8) | -4.1 (-11.3, 3.8) | 0.281 |
|  | Lag4 | 3.0 (-2.7, 9.2) | 1.3 (-3.6, 6.4) | 0.633 | |  | 1.8 (-5.2, 9.4) | 2.5 (-4.6, 10.1) | 0.889 |
|  | 5dMA | -1.9 (-10.3, 7.3) | 1.6 (-6, 9.9) | 0.500 | |  | -12 (-23.7, 1.4) | -5.4 (-15.6, 6.1) | 0.312 |
| QTc | Lag0 | 0.5 (-0.2, 1.1) | 0.5 (-0.1, 1.1) | 0.875 | |  | 0.7 (-0.2, 1.6) | 0.4 (-0.4, 1.2) | 0.578 |
|  | Lag1 | 0.1 (-0.5, 0.8) | 0.0 (-0.6, 0.6) | 0.741 | |  | 0.4 (-0.5, 1.3) | -0.2 (-1.0, 0.5) | 0.213 |
|  | Lag2 | 0.2 (-0.4, 0.8) | 0.1 (-0.4, 0.6) | 0.901 | |  | 0.1 (-0.7, 1.0) | 0.0 (-0.8, 0.7) | 0.732 |
|  | Lag3 | 0.3 (-0.3, 0.9) | 0.2 (-0.3, 0.8) | 0.869 | |  | 0.3 (-0.5, 1.2) | -0.2 (-1.0, 0.6) | 0.321 |
|  | Lag4 | 0.4 (-0.2, 0.9) | -0.2 (-0.7, 0.3) | 0.101 | |  | 0.4 (-0.3, 1.1) | -0.2 (-0.9, 0.5) | 0.226 |
|  | 5dMA | 0.6 (-0.3, 1.6) | 0.4 (-0.4, 1.2) | 0.602 | |  | 1.2 (-0.3, 2.7) | 0.1 (-1.1, 1.3) | 0.160 |
| T Complexity | Lag0 | 2.5 (-4.4, 9.8) | 0.7 (-5.4, 7.3) | 0.687 | |  | -2.1 (-10.7, 7.4) | 4.7 (-3.8, 14.0) | 0.222 |
|  | Lag1 | 4.0 (-3.0, 11.5) | 1.6 (-4.7, 8.2) | 0.566 | |  | -7.7 (-15.7, 1.2) | 2.3 (-5.3, 10.5) | 0.061 |
|  | Lag2 | 3.2 (-3.0, 9.8) | -3.2 (-8.4, 2.2) | 0.097 | |  | -2.6 (-11.0, 6.7) | 0.2 (-7.5, 8.6) | 0.609 |
|  | Lag3 | 4.0 (-2.2, 10.5) | -0.9 (-6.2, 4.7) | 0.222 | |  | -2.2 (-10.2, 6.5) | -0.1 (-8.4, 8.9) | 0.704 |
|  | Lag4 | 0.8 (-5.3, 7.2) | -0.4 (-5.5, 5.1) | 0.763 | |  | -1.9 (-9.1, 5.9) | 4.4 (-3.3, 12.7) | 0.240 |
|  | 5dMA | 7.9 (-2.1, 18.9) | 0.0 (-8.1, 8.9) | 0.189 | |  | -6.0 (-19.5, 9.8) | 4.7 (-7.6, 18.7) | 0.170 |

Note: the effect estimates of biomarkers per IQR increase in PM_2.5_ or O_3_ were adjusted by NO_2_. * *p* < 0.05 for significant association within a group.

**Supplemental Table 9**. Percent change (95% CI) in biomarkers per IQR increase in PM_2.5_ and O_3_ in the low and high omega-3 groups after excluding outliers.

| Outcome | Lag (Day) | PM_2.5_ | | |  | O_3_ | | |
| --- | --- | --- | --- | --- | --- | --- | --- | --- |
|  |  | Low | High | *p*_interaction_ |  | Low | High | *p*_interaction_ |
| Triglycerides | Lag0 | -1.1 (-8.4, 6.7) | 3.3 (-3.7, 10.8) | 0.380 |  | 3.1 (-6.7, 13.9) | 3.5 (-5.5, 13.3) | 0.944 |
|  | Lag1 | -0.7 (-7.3, 6.5) | 8.1 (1, 15.8) ^*^ | 0.073 |  | 5 (-5.3, 16.4) | 0.4 (-8.3, 9.8) | 0.465 |
|  | Lag2 | -2.8 (-9.1, 4) | -3 (-8.8, 3.2) | 0.959 |  | 3 (-6.8, 13.9) | -4.2 (-12.7, 5.1) | 0.239 |
|  | Lag3 | -0.3 (-6.8, 6.7) | -5.4 (-11.1, 0.6) | 0.243 |  | 0.8 (-8.6, 11.1) | -7.1 (-15.9, 2.6) | 0.197 |
|  | Lag4 | -4.5 (-10.9, 2.3) | -0.8 (-6.5, 5.2) | 0.400 |  | 2.3 (-6.4, 11.7) | -7.7 (-15.4, 0.7) | 0.087 |
|  | 5dMA | -4.5 (-14, 6.1) | -0.6 (-9.8, 9.5) | 0.558 |  | 5.8 (-11, 25.8) | -5.2 (-18.1, 9.7) | 0.206 |
| tPA | Lag0 | -3.7 (-9.3, 2.3) | 1.5 (-3.4, 6.6) | 0.161 |  | -0.3 (-7.2, 7.2) | 0.1 (-6.4, 7) | 0.939 |
|  | Lag1 | -3.5 (-8.7, 2) | -0.2 (-4.9, 4.7) | 0.332 |  | 1.7 (-5.7, 9.7) | -1.1 (-7.3, 5.4) | 0.532 |
|  | Lag2 | 3.9 (-1, 8.9) | -0.1 (-4.4, 4.3) | 0.221 |  | 1.8 (-5.5, 9.5) | 1.1 (-5.3, 8) | 0.892 |
|  | Lag3 | 4.8 (-0.4, 10.2) | 1.6 (-2.7, 6.1) | 0.354 |  | 3.4 (-3.7, 11) | 4.2 (-3.1, 12) | 0.862 |
|  | Lag4 | -2.8 (-7.7, 2.3) | 1.3 (-2.9, 5.6) | 0.212 |  | -0.3 (-6.5, 6.2) | 1.6 (-4.5, 8) | 0.661 |
|  | 5dMA | 0.1 (-7.7, 8.6) | 2.1 (-4.6, 9.4) | 0.691 |  | 4.2 (-8, 18.2) | 3.8 (-6.7, 15.4) | 0.947 |
| D-dimer | Lag0 | 5.7 (-2.7, 14.8) | -0.9 (-8.2, 6.8) | 0.230 |  | 5 (-6.2, 17.6) | 6.1 (-4, 17.2) | 0.885 |
|  | Lag1 | 9.5 (1.4, 18.2) ^*^ | 2.2 (-5.1, 10.1) | 0.186 |  | 4.5 (-6.8, 17.2) | 2 (-7.6, 12.5) | 0.719 |
|  | Lag2 | 7.3 (-0.3, 15.5) | 1.3 (-5.3, 8.2) | 0.231 |  | 1.9 (-8.9, 14) | -0.1 (-9.7, 10.4) | 0.761 |
|  | Lag3 | 5.3 (-2.5, 13.7) | 1 (-5.6, 8.2) | 0.413 |  | 2.3 (-8.2, 14.1) | 9 (-2.2, 21.5) | 0.365 |
|  | Lag4 | -2.3 (-9.6, 5.6) | -1 (-7.3, 5.7) | 0.797 |  | -5.8 (-14.6, 3.9) | 5.9 (-3.6, 16.4) | 0.073 |
|  | 5dMA | 12.1 (-0.2, 25.9) | 1.9 (-8.5, 13.5) | 0.202 |  | 6.5 (-12.1, 28.9) | 11 (-5.3, 30.1) | 0.665 |
| SAA | Lag0 | 2.5 (-11.4, 18.5) | 0.5 (-12.1, 14.8) | 0.836 |  | 3.6 (-14.4, 25.5) | 1.4 (-14.8, 20.8) | 0.852 |
|  | Lag1 | 6.8 (-6.6, 22.1) | -5.2 (-16.6, 7.7) | 0.185 |  | 11.8 (-8.9, 37.2) | 2.5 (-13.4, 21.4) | 0.471 |
|  | Lag2 | -1.9 (-13.5, 11.3) | -1.6 (-12.1, 10.3) | 0.970 |  | 7.3 (-11.7, 30.3) | -8.4 (-23, 9) | 0.181 |
|  | Lag3 | 4.2 (-8.3, 18.5) | -5 (-15.7, 7) | 0.278 |  | 10 (-9, 33.1) | 7.3 (-11.7, 30.4) | 0.836 |
|  | Lag4 | 14.3 (0.1, 30.4) ^*^ | 2.3 (-8.4, 14.3) | 0.196 |  | 5.1 (-11.4, 24.5) | 6.3 (-9.7, 25.2) | 0.916 |
|  | 5dMA | 11.2 (-8.8, 35.4) | -4.8 (-21, 14.8) | 0.221 |  | 25.9 (-10.5, 77.2) | 9.8 (-17.3, 45.9) | 0.414 |
| sICAM-1 | Lag0 | 0.5 (-2.1, 3.1) | 1 (-1.4, 3.4) | 0.773 |  | 4.8 (1.3, 8.5) ^*^ | -0.1 (-3.1, 3) | 0.022 |
|  | Lag1 | 0.6 (-1.9, 3) | 2.1 (-0.2, 4.5) | 0.348 |  | 2.9 (-0.8, 6.6) | 0.2 (-2.8, 3.3) | 0.227 |
|  | Lag2 | -1.5 (-3.8, 0.8) | 0.2 (-1.9, 2.3) | 0.265 |  | 0.5 (-3, 4.1) | 0.6 (-2.6, 3.8) | 0.969 |
|  | Lag3 | 0.3 (-2, 2.7) | -0.4 (-2.5, 1.8) | 0.648 |  | -1 (-4.4, 2.6) | -2.8 (-6.1, 0.7) | 0.406 |
|  | Lag4 | 0.2 (-2.3, 2.7) | -0.7 (-2.7, 1.3) | 0.566 |  | -0.4 (-3.5, 2.8) | -3 (-5.8, -0.1) | 0.208 |
|  | 5dMA | -0.1 (-3.7, 3.6) | 0.6 (-2.7, 4.1) | 0.748 |  | 3 (-3.3, 9.7) | -2.2 (-7.1, 3) | 0.097 |
| sVCAM-1 | Lag0 | 0.7 (-2, 3.5) | 0.8 (-1.7, 3.3) | 0.983 |  | 2.5 (-1.2, 6.4) | -2.3 (-5.4, 1) | 0.034 |
|  | Lag1 | 0.9 (-1.6, 3.6) | 1.2 (-1.2, 3.8) | 0.858 |  | 2.1 (-1.6, 6.1) | -0.6 (-3.8, 2.7) | 0.238 |
|  | Lag2 | 0.4 (-2, 2.9) | -0.4 (-2.6, 1.8) | 0.591 |  | 0.9 (-2.8, 4.6) | -0.7 (-4, 2.6) | 0.477 |
|  | Lag3 | 1.9 (-0.6, 4.5) | -0.6 (-2.8, 1.6) | 0.124 |  | -1.9 (-5.3, 1.7) | -3 (-6.5, 0.6) | 0.628 |
|  | Lag4 | 1.3 (-1.3, 3.8) | -0.9 (-3, 1.2) | 0.187 |  | -0.9 (-4.1, 2.3) | -2.1 (-5.1, 1) | 0.594 |
|  | 5dMA | 2.4 (-1.4, 6.4) | -0.1 (-3.5, 3.4) | 0.311 |  | 0 (-6.1, 6.5) | -3.9 (-8.8, 1.3) | 0.221 |

| Supplemental Table 8 continued | | | | | | | | |
| --- | --- | --- | --- | --- | --- | --- | --- | --- |
| SDNN | Lag0 | -1.2 (-6, 3.9) | -2 (-6.4, 2.7) | 0.805 |  | 0.4 (-6.1, 7.4) | 0.9 (-5, 7.1) | 0.917 |
|  | Lag1 | -1 (-5.6, 3.8) | -3 (-7.3, 1.5) | 0.503 |  | 1.2 (-5.6, 8.5) | -2.1 (-7.9, 4.1) | 0.432 |
|  | Lag2 | 3.8 (-0.8, 8.6) | -1 (-4.9, 3.1) | 0.110 |  | 1.4 (-5.3, 8.6) | -2.1 (-7.8, 4) | 0.405 |
|  | Lag3 | 3.1 (-1.4, 7.9) | -0.6 (-4.6, 3.5) | 0.217 |  | 2 (-4.4, 8.8) | -1.9 (-8.2, 4.7) | 0.347 |
|  | Lag4 | 2.2 (-2.4, 7) | -1 (-4.8, 3) | 0.291 |  | 2.5 (-3.4, 8.7) | 3.9 (-1.8, 9.9) | 0.725 |
|  | 5dMA | 3.3 (-3.7, 10.8) | -3 (-9.2, 3.5) | 0.157 |  | 5.5 (-6.2, 18.6) | 1.1 (-8.2, 11.3) | 0.472 |
| RMSSD | Lag0 | -3.7 (-10.8, 4) | -1.2 (-8.1, 6.1) | 0.616 |  | -2 (-11.6, 8.6) | -0.7 (-9.4, 8.9) | 0.826 |
|  | Lag1 | -1.2 (-8.1, 6.2) | -1.4 (-8, 5.7) | 0.980 |  | 4.4 (-6.1, 16.1) | 0.4 (-8.5, 10.1) | 0.534 |
|  | Lag2 | 3.7 (-3.2, 11.1) | -0.2 (-6.2, 6.2) | 0.394 |  | 1.7 (-8.4, 12.8) | -0.9 (-9.6, 8.7) | 0.694 |
|  | Lag3 | 2.7 (-4.1, 10) | -0.9 (-7, 5.6) | 0.436 |  | 5 (-4.9, 15.9) | 0.4 (-9.3, 11.1) | 0.487 |
|  | Lag4 | 1.3 (-5.5, 8.7) | -0.3 (-6.1, 5.9) | 0.720 |  | 3.4 (-5.4, 13.1) | 7.7 (-1.2, 17.4) | 0.503 |
|  | 5dMA | 1.5 (-8.9, 13) | -1.5 (-10.8, 8.9) | 0.667 |  | 10.3 (-7.7, 32) | 5.8 (-8.6, 22.6) | 0.645 |
| LF/HF | Lag0 | 8.4 (-5, 23.6) | 3.8 (-8.2, 17.4) | 0.617 |  | -0.5 (-16.6, 18.7) | -9.9 (-23.3, 5.9) | 0.352 |
|  | Lag1 | 1.1 (-10.6, 14.4) | 2.5 (-9, 15.5) | 0.866 |  | -7.8 (-23.1, 10.6) | -3.9 (-17.8, 12.3) | 0.706 |
|  | Lag2 | 2.7 (-8.9, 15.7) | 4.6 (-5.8, 16.2) | 0.805 |  | 8.1 (-9.7, 29.4) | 3.8 (-11.3, 21.6) | 0.713 |
|  | Lag3 | 0.7 (-10.6, 13.5) | 1.8 (-8.5, 13.3) | 0.888 |  | 3.4 (-12.6, 22.4) | -11 (-24.9, 5.4) | 0.168 |
|  | Lag4 | 1.6 (-9.9, 14.6) | -3.7 (-12.9, 6.5) | 0.496 |  | 0.4 (-13.9, 17) | -9.3 (-21.6, 5) | 0.324 |
|  | 5dMA | 6.4 (-11.5, 28) | 4.7 (-11.4, 23.7) | 0.885 |  | -3 (-28.6, 31.8) | -12.4 (-31.7, 12.5) | 0.509 |
| VLF | Lag0 | -21.2 (-35.2, -4.2) ^*^ | -3.1 (-19.2, 16.3) | 0.107 |  | -19.9 (-38.8, 4.8) | 3.3 (-19.1, 31.8) | 0.109 |
|  | Lag1 | -7.1 (-22.9, 11.9) | -7.2 (-22.4, 10.9) | 0.991 |  | 4.2 (-20.8, 37.3) | 9.2 (-13.5, 37.9) | 0.771 |
|  | Lag2 | 1.2 (-15.7, 21.3) | -8.7 (-22.4, 7.5) | 0.395 |  | 16.6 (-10.9, 52.7) | 4.1 (-18, 32) | 0.483 |
|  | Lag3 | 10.5 (-7.8, 32.3) | 11.7 (-5.2, 31.5) | 0.928 |  | 8.6 (-15.9, 40.3) | -10.7 (-30.6, 14.9) | 0.227 |
|  | Lag4 | 1.4 (-15.4, 21.7) | 2.8 (-12.3, 20.6) | 0.911 |  | -2.2 (-22.4, 23.2) | -0.7 (-20.8, 24.5) | 0.920 |
|  | 5dMA | -6 (-28.8, 24.1) | -2.4 (-24.8, 26.5) | 0.833 |  | 2.4 (-35.6, 62.9) | 1.8 (-30.3, 48.8) | 0.979 |
| P Complexity | Lag0 | 2.9 (-3.8, 10.2) | 2.7 (-3.4, 9.3) | 0.964 |  | -0.8 (-9.7, 9) | 1.2 (-6.9, 9.9) | 0.720 |
|  | Lag1 | 1.6 (-4.7, 8.3) | 5 (-1.2, 11.5) | 0.437 |  | 0.2 (-8.7, 10.1) | 0.1 (-7.5, 8.4) | 0.983 |
|  | Lag2 | 4.6 (-1.7, 11.3) | 5.3 (-0.3, 11.2) | 0.878 |  | -1.7 (-10.4, 7.8) | -2.6 (-10.2, 5.7) | 0.868 |
|  | Lag3 | 4.7 (-1.6, 11.4) | 2.5 (-3, 8.3) | 0.604 |  | -1.7 (-10.1, 7.6) | 0.6 (-8, 10.1) | 0.690 |
|  | Lag4 | -0.9 (-6.8, 5.4) | 7.6 (2.2, 13.3) ^*^ | 0.039 |  | -1.4 (-8.9, 6.8) | 8.5 (0.5, 17.1) ^*^ | 0.076 |
|  | 5dMA | 6.8 (-2.9, 17.5) | 12.4 (3.2, 22.5) ^*^ | 0.388 |  | -1.7 (-16.3, 15.4) | 3.4 (-9.3, 18) | 0.528 |
| T Complexity | Lag0 | 3.2 (-3.2, 9.9) | 1.2 (-4.4, 7.2) | 0.643 |  | -2.4 (-10.5, 6.5) | 5.1 (-2.8, 13.6) | 0.159 |
|  | Lag1 | 5.6 (-0.6, 12.2) | 3.8 (-2, 10.1) | 0.677 |  | -5.8 (-13.8, 3.1) | 3.1 (-4.5, 11.3) | 0.094 |
|  | Lag2 | 3.7 (-2.2, 9.9) | -2.8 (-7.7, 2.4) | 0.094 |  | -1.8 (-10.1, 7.2) | 1.4 (-6.2, 9.6) | 0.546 |
|  | Lag3 | 3.4 (-2.4, 9.5) | -1.3 (-6.6, 4.2) | 0.231 |  | -1.9 (-9.7, 6.6) | 1.2 (-7.1, 10.2) | 0.563 |
|  | Lag4 | -1 (-6.6, 5.1) | -0.6 (-5.5, 4.6) | 0.929 |  | -2.2 (-9.3, 5.6) | 3.9 (-3.5, 11.7) | 0.246 |
|  | 5dMA | 6.2 (-3, 16.3) | 0.4 (-7.6, 9.1) | 0.326 |  | -6.1 (-19.3, 9.2) | 5.6 (-6.8, 19.7) | 0.127 |

Note: this table only presents outcomes that have outliers excluded. * *p* < 0.05 for significant association within a group.
